# Associations between Dietary Pattern Networks Derived from Machine Learning Algorithms and Cardiovascular Disease Risk in the NutriNet-Santé Cohort

**DOI:** 10.1016/j.tjnut.2025.09.014

**Published:** 2025-09-23

**Authors:** Mélina Côté, Joy M Hutchinson, Mathilde Touvier, Bernard Srour, Laurent Bourhis, Benoît Lamarche, Léopold K Fezeu

**Affiliations:** 1 Centre Nutrition, Santé et Société (NUTRISS), Institut sur la Nutrition et les Aliments Fonctionnels (INAF), Université Laval, Québec, Québec, Canada; 2 École de Nutrition, Université Laval, Québec, Québec, Canada; 3 Université Sorbonne Paris Nord and Université Paris Cité, INSERM, INRAE, CNAM, Centre of Research in Epidemiology and StatisticS (CRESS), Nutritional Epidemiology Research Team (EREN), Bobigny, France

**Keywords:** Gaussian graphical model, Louvain algorithm, dietary patterns, NutriNet-Santé, cardiovascular incidence

## Abstract

**Background:**

Major advances in the fields of data science and machine learning have enabled the use of novel methods, such as Gaussian graphical models (GGMs) and the Louvain algorithm, to identify dietary patterns (DP).

**Objectives:**

The aim of this study was to identify DP networks using novel computational approaches and to investigate the associations between these DP networks and cardiovascular disease (CVD) risk in a sample of the French population.

**Methods:**

A sample of 99,362 participants aged ≥15 y from the NutriNet-Santé cohort was used. Dietary intakes (reported as grams per day) were assessed using ≥2 24-h dietary records, which were then classified into 42 food groups. CVD events were assessed using health questionnaires and subsequently validated based on medical records. GGMs were employed with the Louvain algorithm to derive DP networks. GGMs are network models that depict relationships among many variables (food groups) based on conditional correlation matrices. The Louvain algorithm extracts nonoverlapping communities from large networks. The relationship between DP networks and CVD incidence was evaluated using proportional hazard Cox models, adjusted for confounding variables.

**Results:**

Analyses revealed 5 distinct DP networks reflecting consumption of *1*) appetizer foods, *2*) breakfast foods, *3*) plant-based foods, *4*) ultraprocessed sweets and snacks, and *5*) healthy foods. Among these, only the DP network of ultraprocessed sweets and snacks was associated with greater CVD risk when adjusted for energy and potential confounders including overall diet quality (hazard ratio of quintile 5 compared with quintile 1: 1.32; 95% confidence interval: 1.11, 1.57; *P*-trend = 0.0002).

**Conclusions:**

The results suggest that a DP network reflecting the consumption of ultraprocessed sweets and snacks is associated with incident CVD in a sample of the French population, independent of diet quality. The innovative approach to derive empirical DP networks may assist in the identification of food groups that are likely to be consumed together in a population, thereby helping to identify dietary habits to target for the prevention of CVD.

This trial was registered at clinicaltrials.gov as NCT03335644.

## Introduction

In recent decades, nutrition research has undergone a significant shift in focus, moving away from the study of individual nutrients or foods toward the examination of dietary patterns [1]. This shift has been driven by the recognition of the intricate and interdependent relationships between foods and the need to understand their collective impact on health [[2], [3], [4]]. A number of dietary patterns have been identified based on a priori knowledge, including the Dietary Approaches to Stop Hypertension diet and the Mediterranean diet [5]. Furthermore, various exploratory data-driven methods have been employed to derive dietary patterns, including principal component analysis [[6], [7], [8]], reduced rank regression [[9], [10], [11]], cluster analysis [8,12], and factor analysis [7,8]. These data-driven approaches have revealed multiple dietary patterns that are associated with health outcomes such as cancer, obesity, type 2 diabetes, hypertension, and cardiovascular diseases (CVDs) [[6], [7], [8], [9], [10],[13], [14], [15]].

In light of the rapid advances in the fields of data science and machine learning, new and more complex methods have been developed to identify dietary patterns associated with health outcomes [[16], [17], [18], [19], [20], [21]]. Among these innovative methods, Gaussian graphical models (GGMs) combined with the Louvain algorithm represent novel graph theory-based methods [22] that yield complex networks reflecting a variety of dietary patterns. GGMs depict relationships among a vast array of variables, such as food groups, based on pairwise partial correlations that are adjusted for the indirect associations of all other variables in the model. This analysis enables the inference of conditional independence among variables and the identification of internal patterns within a given dataset. GGMs have been employed to extract dietary pattern networks among specific populations, including pregnant females and individuals with abdominal obesity [[23], [24], [25]]. Additionally, GGMs have been used to identify dietary pattern networks associated with health outcomes [[26], [27], [28]]. For example, Gunathilake et al. [27] identified 4 dietary pattern networks using GGMs and demonstrated that 2 of these networks were associated with a reduced risk of gastric cancer in a Korean population. The Louvain algorithm is an unsupervised community detection algorithm used to identify nonoverlapping communities within GGM-generated networks. The algorithm is based on modularity optimization in which variables (referred to as nodes) are iteratively clustered together until the optimal grouping of nodes is reached [29]. The Louvain algorithm is being increasingly employed in nutrition and healthcare studies [[30], [31], [32]]. For example, Perraud et al. [31] demonstrated how graph theory and the Louvain algorithm can be employed to identify dietary habit changes that require prioritization when transitioning toward a healthy diet.

To our knowledge, the combination of GGMs and the Louvain algorithm has not yet been employed to identify empirically derived dietary pattern networks and their relationships with incident CVDs. Hence, the purpose of this study was 2-fold. We first identified dietary pattern networks using the combination of GGMs and the Louvain algorithm in a large population-based French cohort. We then examined the prospective associations between the identified dietary pattern networks and incident CVD in this population.

## Methods

### Study population and data collection

The NutriNet-Santé study is a prospective, web-based cohort study that commenced in 2009 in France. The study design and methodology have been previously described [33]. In brief, the objective of the NutriNet-Santé study is to examine the relationships between nutrition and health in the French population over time. The open and ongoing recruitment of the NutriNet-Santé study targets participants aged ≥15 y who speak French and have Internet access. On enrollment, participants are required to complete online questionnaires documenting dietary habits, health status, anthropometrics, lifestyle habits, sociodemographic characteristics, and physical activity levels. All participants provided informed consent. The study was registered at clinicaltrials.gov (NCT03335644) and is being conducted in accordance with the Declaration of Helsinki. It was approved by the Institutional Review Board of the French Institute for Health and Medical Research (IRB-Inserm) and the Commission Nationale de l’Informatique et des Libertés (CNIL No. 908450/909216).

### Dietary assessment

On enrollment, participants included in this study were invited to complete 3 24-h dietary records, administered randomly on nonconsecutive days (2 weekdays and 1 weekend day) over a 2-wk period. This was repeated every 6 mo thereafter. Participants were required to indicate all foods and beverages consumed over a 24-h period, along with quantities consumed. The 24-h dietary records administered in the NutriNet-Santé study have previously been validated in the French population using interviewer-generated food records and blood and urine biomarkers as references [[34], [35], [36]]. To facilitate the present analyses, dietary intakes were averaged using all 24-h dietary records completed over the first 2 y of participation. All foods reported (in grams per day) in the dietary records were classified into 42 food groups based on food categories determined by the NutriNet-Santé team. Foods that could not be categorized in any of the groups (e.g., ingredients such as flour) were excluded.

Overall diet quality was assessed using 2 approaches. First, the Programme National Nutrition Santé guidelines score 2 (PNNS 2 score) was calculated. The score, which was developed and validated in the French population, ranges from −17 to 13.5 with higher scores reflecting greater adherence to the French dietary guidelines of 2017 [37]. Second, intake of ultraprocessed foods (UPFs) as defined according to the NOVA food classification system was calculated as the proportion of total energy intake coming from the NOVA-4 foods [38,39]. This variable is henceforth referred to as the %UPF.

### CVD assessment

The occurrence of CVD events was first assessed through the administration of health questionnaires to the NutriNet-Santé participants on a biannual basis. Additionally, participants were afforded the opportunity to report any changes in their health status via the NutriNet-Santé Web interface at any time. All self-reported CVD events were validated through the relevant medical records collected by the study’s physicians, by the patient’s physician, or by the hospital providing treatment. All information was subjected to review by a committee of study doctors to validate major health events. Those participants who had been inactive on the study website for a period >1 y were contacted by the team of study doctors to ascertain whether they had experienced a recent CVD event or other major health problem. Finally, data were paired with the medico-administrative databases of the national health insurance system (SNIIRAM) and the national mortality registry (CépiDC) to further limit potential biases related to participants not reporting CVD events.

CVD events were classified in accordance with the International Classification of Diseases, 10th Revision. The present analyses included fatal and nonfatal coronary artery disease (acute coronary syndrome, code I21.4; myocardial infarction, code I21; angina pectoris, code I20.0; angioplasty, code Z95.8) and cerebrovascular disease (stroke, code I64; transient ischemic attack, codes G45.8 and G45.9). All CVD events diagnosed between the date of inclusion and 30 September, 2022 were included in the present analyses.

### Statistical analyses

For the purposes of the present analyses, the baseline period was defined as the 2-y period following the participants’ enrollment in the NutriNet-Santé study. Individuals who were inactive on the NutriNet-Santé interface since enrollment, who had missing health data, who had experienced a CVD event before enrollment or during the first 2 y of participation, who underreported energy intakes [40], who completed fewer than 2 24-h dietary records, or who were pregnant were excluded from the analyses.

#### Dietary pattern network derivation

The GGM combined with the Louvain algorithm was used to identify empirically derived dietary patterns. This approach permitted the mapping of the interrelationships among food groups that underpin the dietary pattern networks in this cohort. Subsequently, dietary pattern network scores were generated to assess the degree of adherence of each individual to each dietary pattern.

##### Gaussian graphical models

GGMs are probabilistic graphs that permit the analysis of conditional dependency structures and the visualization of the internal structure of a dataset [23,41,42]. The conditional independence between 2 variables is inferred using pairwise partial correlations, which are adjusted for the associations with and among all other variables. If the partial correlation between 2 variables is equal to 0, these variables are considered conditionally independent. GGMs are represented as undirected graphs, with each node corresponding to a variable and edges between nodes representing the partial correlation between 2 variables.

In the present study, the term “nodes” was defined a priori as the 42 food groups. Pairwise partial correlations were computed using an inverse covariance matrix (precision matrix) adjusted for all other food groups. In high-dimensional contexts, there may be a limited number of zero entries in the precision matrix. Consequently, the graphical Lasso model, a regularization technique, was employed to enhance the sparsity of the precision matrix. The graphical Lasso model is most effective when applied to data that is normally distributed and standardized. Therefore, all food group variables were transformed using the log function to improve normality and standardized to scale the data. A GGM was generated using the precision matrix to provide a visual representation of all nonzero partial correlations among food groups. The Scikit-learn package [43] in Python 3.9.13 was used to compute the precision matrix, whereas the Networkx package [44] was utilized to visualize the GGM. For technical details on the implementation of GGMs, see Supplemental Appendix 1.

##### Louvain algorithm

The Louvain algorithm is a method employed to identify community structures in graphical networks [29]. This approach is based on optimizing modularity, a metric that quantifies how densely connected the food groups are within a given community compared with what would be expected by chance. For example, a high modularity score indicates that food groups within a particular community are more densely connected to each other (i.e., multiple and strong partial correlations) than to food groups not assigned to this community. In essence, each food group is initially allocated to its own community. Subsequently, neighboring food groups are grouped together to increase the overall modularity. This process is repeated until communities have been formed with the maximum possible modularity, yielding the most meaningful groupings of food groups. In the present analyses, the Louvain algorithm was applied to the GGM-generated network to iteratively search for the partitioning of the network that maximizes modularity and thus identifies the most coherent and interpretable communities within the sample, such that each community reflects a set of food groups that tend to co-occur in individual self-reported diets. The connections among food groups within a community are dense, whereas connections among food groups in different communities are sparse. Robustness of the identified communities was assessed by comparing them with 99 alternative Louvain runs. The normalized mutual information (NMI) measure of agreement, a common metric ranging from 0 (no similarity) to 1 (identical community assignments), was used to quantify the similarity between community structure across runs. Higher NMI values indicate greater consistency, supporting the stability of the detected community structure. The communities had a mean NMI of 0.87 when compared with 99 alternative Louvain algorithm runs, indicating that the overall community structure was consistent across runs and robust to changes in algorithm initialization. The Networkx package in Python 3.9.13 was used to perform this analysis. For technical details on the implementation of the Louvain algorithm, see Supplemental Appendix 1.

##### Dietary pattern network scores

The dietary pattern network scores were calculated according to a method that has been previously employed to investigate the association between dietary pattern networks and health outcomes [28]. This dietary pattern score is based on eigenvector centrality, which is a measure of the relative importance, or “influence,” of each food group within the network. A high eigenvector centrality score for a food group indicates that it has multiple and strong partial correlations with other food groups, thereby reflecting strong integration into the overall dietary pattern represented by the community. These “central” foods may be representative elements of common eating patterns within a population. Unlike simple connectivity measures that count the number of direct links, eigenvector centrality also considers the importance of the food group in relation to the other food groups in the network. In other words, a food group has a high eigenvector centrality value if it is connected to other well-connected food groups. For example, consider a food group A that is connected to several other food groups, which are themselves connected to many other food groups in the network, and a food group B that is connected to several other food groups, which are themselves not connected to other food groups. Food group A would receive a high eigenvector centrality score because it is part of a highly interconnected and “influential” region of the network, and food group B would receive a lower eigenvector centrality score. This reflects not just how many connections a food group has but also the centrality or “influence” of its connections. Each food group within the network has a unique eigenvector centrality value, with standardized values ranging between 0 and 1. To assess the association between the Louvain algorithm-derived dietary patterns and risk of CVD, individual dietary pattern scores were calculated using the eigenvector centrality value assigned to each food group (Supplemental Table 1) multiplied by the individual’s daily intake of that food group (in grams). The dietary pattern network scores for each individual were calculated as the sum of each of these products within each community. The dietary pattern score is therefore influenced not only by the eigenvector centrality of a given food group but also by the quantities of foods consumed by individuals. Higher dietary pattern network scores indicated a higher degree of adherence to that particular dietary pattern.

The statistical significance of the differences in dietary pattern network scores among different subgroups of participants was evaluated using *t* tests or analysis of variance. Pearson correlations were used to assess the associations among dietary pattern network scores and diet quality indices (PNNS 2 score and %UPF).

#### Associations between dietary pattern networks and CVD incidence

The relationship between the dietary pattern network scores and CVD incidence was investigated using multivariable-adjusted proportional hazard Cox models. The chronological age of the participants was used as the time scale in the survival analysis. This was measured from the date of inclusion until the date of the CVD event, the date of the last follow-up, the date of death, or the end of follow-up (30 September, 2022), whichever occurred first.

Cox models were adjusted for variables that have been previously identified as being associated with diet and CVD risk. Model 1 was adjusted for energy intake (in kilocalories per day). Model 2 was further adjusted for sex (male or female), marital status (single, living as a couple or married, divorced, widowed), education level (less than high school degree, less than or equal to a bachelor’s degree, greater than or equal to a bachelor’s degree), smoking status (never smoked, former smoker, current smoker), the number of cigarettes smoked per year (continuous), physical activity level (low, moderate, high), BMI (<18.5 kg/m^2^, 18.5–24.9 kg/m^2^, 25.0–29.0 kg/m^2^, ≥30.0 kg/m^2^), household monthly income (<1100 €/mo, 1100<2300 €/mo, 2300 to <4800 €/month, ≥4800 €/mo), employment status (unemployed, employed, intermediate staff, managerial staff, other), family history of CVD (yes or no), and number of 24-h dietary records (continuous). Finally, model 3 was further adjusted for variations in the scores of the remaining 4 dietary patterns to isolate the independent association of each pattern with CVD. All baseline covariates were represented by the mean of values measured over the 2-y baseline period. Any missing data for the covariates were imputed using the sample mode (<5% of values missing across all covariates included in the models). The proportional hazards assumption was verified using the rescaled Schoenfeld residual method.

The sensitivity analyses included the following: proportional hazard Cox model 2 further adjusted for diet quality indices (PNNS 2 score and %UPF); assessment of potential nonlinear associations between dietary pattern network scores and CVD incidence using a restricted cubic spline transformation with 3 knots (10, 50, 90) or 4 knots (5, 35, 65, 90) for all dietary pattern network scores [45]; models considering hard CVD events only (i.e., fatal or nonfatal acute coronary syndrome, myocardial infarction, and stroke); and models that excluded participants with prevalent type 2 diabetes, hypertension, hypercholesterolemia, and hypertriglyceridemia at baseline. All analyses were conducted using SAS Studio (version 3.81).

## Results

**FIGURE 1:**
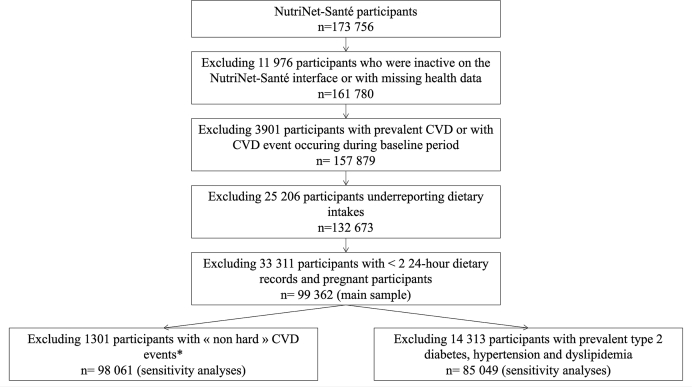
Study flowchart. ∗Sensitivity analysis including only participants with “hard” CVD events, i.e., fatal or nonfatal acute coronary syndrome, myocardial infarction, and stroke. Excluding incident fatal or nonfatal angina pectoris, angioplasty, and transient ischemic attack ensured the reliability of the results. CVD, cardiovascular disease.

A total of 99,362 participants met inclusion criteria among the 173,756 participants enrolled in the NutriNet-Santé cohort at the time of the analysis (Figure 1). Table 1 presents the characteristics of the participants included in the study. The mean age at baseline was 43.1 ± 14.6 y. The majority of participants were females and had a high socioeconomic status at baseline. The mean number of dietary records completed by the participants during the 2-y baseline assessment was 6.1 ± 3.1. Over the 13-y follow-up period, the incidence of CVD was observed in 1.9% of participants (*n* = 1878). The median energy intake in the sample was 1838 kcal/d (IQR: 578) (Supplemental Table 2). The median PNNS 2 score and %UPF in the sample were 1.7 (IQR: 4.73) and 30% (IQR: 20%), respectively.

**TABLE 1:** Baseline characteristics of the sample selected from the NutriNet-Santé cohort (*n* = 99,362).

Characteristic	Value
Age (y), mean ± SD	43.1 ± 14.6
Sex	
Female	78,430 (78.9)
Male	20,932 (21.1)
Education	
<High school degree	17,773 (17.9)
≤Bachelor’s degree	48,817 (49.1)
≥Bachelor’s degree	32,772 (33.0)
Household monthly income, euros/mo	
<1100	6633 (6.7)
1100 to <2300	23,169 (23.3)
2300 to <4800	53,274 (53.6)
≥4800	16,286 (16.4)
Marital status	
Single	19,281 (19.4)
Living as a couple or married	70,291 (70.7)
Divorced or separated	8081 (8.1)
Widowed	1709 (1.7)
Employment status	
Unemployed	37,545 (37.8)
Employed	18,216 (18.3)
Intermediate staff	16,835 (16.9)
Managerial staff	23,450 (23.6)
Farmer, manual worker, or other	3316 (3.3)
BMI (kg/m^2^)	
<18.5	5347 (5.4)
18.5–24.9	64,891 (65.3)
25.0–29.0	20,778 (20.9)
≥30.0	8346 (8.4)
Smoking status	
Never	50,479 (50.8)
Former	32,602 (32.8)
Current	16,281 (16.4)
Physical activity level	
Low	22,464 (22.6)
Moderate	45,679 (46.0)
High	31,219 (31.4)
Number of cigarette packs per year among current smokers (median, Q1–Q3)	3.8, 0.5–11.7
Family history of CVD	63,952 (64.4)
Prevalent type 2 diabetes	1396 (1.4)
Prevalent hypertension	7624 (7.7)
Prevalent hypertriglyceridemia	1528 (1.5)
Prevalent hypercholesterolemia	8020 (8.1)
Number of 24 h dietary records (mean ± SD)	6.1 ± 3.1
Incidence of CVD	1878 (1.9)
	Median (Q1–Q3)
Energy (kcal/d)	1838.4 (1579.0–2157.3)
PNNS 2 score	1.7 (−0.83 to 3.9)
%UPF	30 (20–40)

Values are *n* (%) unless stated otherwise. %UPF represents the proportion of self-reported energy intake as UPF defined based on the NOVA food classification system.

Abbreviations: CVD, cardiovascular disease; PNNS 2 score, Programme National Nutrition Santé guidelines score 2; UPF, ultraprocessed foods.

**FIGURE 2:**
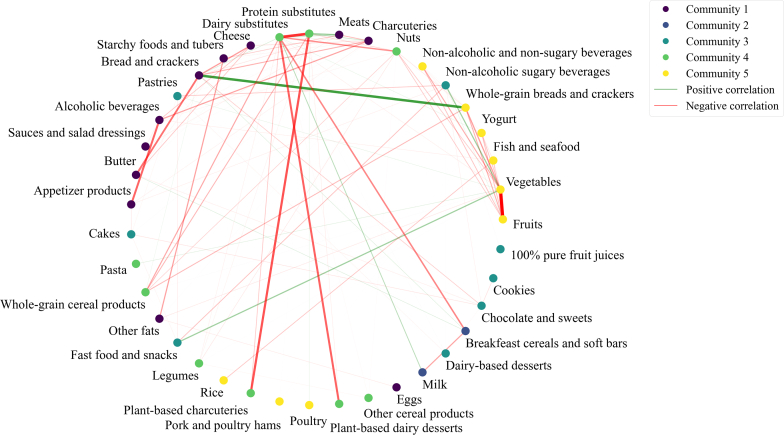
Gaussian graphical model presenting all nonzero partial correlations between food groups, adjusted for all other food groups, and communities identified by the Louvain algorithm. Community 1 “Appetizers” included 11 food groups: alcoholic beverages, appetizer products, breads and crackers, butter, charcuteries, cheese, eggs, meats, other fats, sauces and salad dressings, and starchy foods and tubers. Community 2 “Breakfast” included 2 food groups: breakfast cereals and soft bars, and milk. Community 3 “Sweets and snacks” included 8 food groups: cakes, chocolate and sweets, cookies, dairy-based desserts, fast food and snacks, 100% pure fruit juices, nonalcoholic sugary beverages, and pastries. Community 4 “Plant-based” included 9 food groups: dairy substitutes, legumes, nuts, other cereal products, pasta, plant-based charcuteries, plant-based dairy desserts, protein substitutes, and whole-grain cereal products. Community 5 “Healthy” included 9 food groups: fish and seafood, fruits, nonalcoholic and nonsugary beverages, pork and poultry hams, poultry, rice, vegetables, whole-grain breads and crackers, and yogurts.

Of the 42 food groups, the fish charcuterie, offal, and vegetable juice food groups did not exhibit ≥1 partial correlation that differed from 0 and thus were excluded from the GGM-estimated network. Figure 2 presents the GGM-estimated network of the 39 conditionally dependent food groups (partial correlation different than 0), described through 5 communities identified by the Louvain algorithm. The pairwise conditional correlation coefficients ranged between −0.25 and 0.19 (Supplemental Table 3). Community 1 comprised 11 food groups, including alcoholic beverages, appetizer products, breads and crackers, butter, charcuteries, cheese, eggs, and meats. This community was labeled as “Appetizers.” Community 2 was comprised of breakfast cereals and soft bars and milk and was labeled as “Breakfast.” Community 3 was labeled “Sweets and snacks” based on the 8 food groups that it comprised, namely, cakes, chocolate and sweets, cookies, dairy-based desserts, fast food and snacks, 100% pure fruit juices, nonalcoholic sugary beverages, and pastries. Community 4 consisted primarily of plant-based foods, including plant-based charcuteries, nuts, legumes, and dairy substitutes, and was labeled as “Plant-based.” Finally, community 5 comprised 9 food groups that are generally considered to be part of a healthy diet, including fish and seafood, fruits, nonalcoholic and nonsugary beverages, poultry, rice, vegetables, whole-grain breads and crackers, and yogurts. This community was labeled as “Healthy.”

Table 2 presents the food groups in each of the GGM-generated dietary pattern networks with respect to their contribution to the dietary pattern network scores, the median scores for each dietary pattern network, and their associations with diet quality indices. The scores for community 4 “Plant-based” and community 5 “Healthy” were positively correlated with the PNNS 2 score (*r* = 0.25 and 0.30, respectively), suggesting that these communities represent healthier dietary patterns. Community 3 “Sweets and snacks” was positively correlated with the %UPF (*r* = 0.46), consistent with a dietary pattern network of low-quality foods. Community 1 “Appetizers” was negatively correlated with both the PNNS 2 score (*r* = −0.56), and with %UPF (*r* = −0.16), also reflecting a dietary pattern network of low-quality foods. There was no evidence of collinearity among the dietary pattern network scores (Supplemental Table 4). Supplemental Table 5 presents the differences in dietary pattern network scores among subgroups of the sample.

**TABLE 2:** Food groups included in each dietary pattern network score, median dietary pattern network scores in the sample, and Pearson correlations with diet quality indices.

Dietary pattern network	Community 1 “Appetizers”	Community 2 “Breakfast”	Community 3 “Sweets and snacks”	Community 4 “Plant-based”	Community 5 “Healthy”
Total number of food groups	11	2	8	9	9
	Foods groups included in the dietary pattern network score in order of importance^1^				
	Bread and crackers	Milk	Nonalcoholic sugary beverages	Dairy substitutes	Nonalcoholic and nonsugary beverages
	Alcoholic beverages	Breakfast cereals and soft bars	Chocolate and sweets	Legumes	Vegetables
	Meats		Cakes	Other cereal products	Fruits
	Cheese		Dairy-based desserts	Whole-grain cereal products	Yogurts
	Charcuteries		Fast food and snacks	Nuts	Whole-grain breads and crackers
	Starchy foods and tubers		100% pure fruit juices	Pasta	Fish and seafood
	Sauces and salad dressings		Pastries	Protein substitutes	Poultry
	Butter		Cookies	Plant-based dairy desserts	Pork and poultry hams
	Other fats			Plant-based charcuteries	Rice
	Appetizer products				
	Eggs				
Median (Q1–Q3)	10.8 (7.3–15.4)	0.5 (0.0–2.4)	4.6 (2.8–7.2)	1.0 (0.4–2.1)	40.3 (30.4–51.4)
Pearson correlation coefficients					
PNNS 2 score	−0.56	−0.06	−0.38	0.25	0.30
%UPF	−0.16	0.06	0.46	−0.03	−0.34

Abbreviations:PNNS 2 score, Programme National Nutrition Santé guidelines score 2; UPF, ultraprocessed foods.

%UPF represents the proportion of self-reported energy intake as UPF defined based on the NOVA food classification system.

1 Ranking of food groups in the dietary pattern network score based on the product of eigenvector centrality measures and the mean consumption of that food group in grams per day in the sample (see Methods section, Dietary pattern network derivation).

Table 3 presents the hazard ratios (HRs) of CVD in the sample according to fifths of the dietary pattern network scores, both before and after adjustment for different sets of covariates. The HR for CVD among participants in the top fifth of the community 3 “Sweets and snacks” dietary pattern scores compared with those in the lowest fifth was 34% higher in the fully adjusted model (HR: 1.34; 95% confidence interval [CI]: 1.12, 1.59; *P*-trend = 0.0001). This association remained significant after adjustment for concurrent variations in scores of all other dietary pattern networks (Supplemental Table 6, HR_Q5 vs Q1_: 1.31; 95% CI: 1.09, 1.57; *P*-trend = 0.0004). The dietary pattern network scores of the other 4 communities showed no association with incident CVD in multivariable analyses.

**TABLE 3:** Multivariable associations between adherence to dietary pattern network scores (1 community per model) and cardiovascular disease incidence in a sample of the NutriNet-Santé cohort (*n* = 99,362).

Exposure	Hazard ratio (95% confidence interval)					*P*-trend
Fifths	1	2	3	4	5	
Community 1 “Appetizers”						
Number of participants	19,872	19,873	19,872	19,873	19,872	
Number of cases	234	284	336	394	630	
Model 1	1.00	1.05 (0.88, 1.24)	1.07 (0.90, 1.26)	1.07 (0.90, 1.26)	1.26 (1.06, 1.50)	0.009
Model 2	1.00	1.03 (0.86, 1.22)	1.03 (0.86, 1.22)	0.95 (0.80, 1.13)	0.93 (0.78, 1.12)	0.27
Community 2 “Breakfast”						
Number of participants	27,430	12,262	19,925	19,837	19,908	
Number of cases	494	271	406	364	343	
Model 1	1.00	1.03 (0.89, 1.20)	1.09 (0.95, 1.24)	1.09 (0.95, 1.24)	1.01 (0.88, 1.17)	0.54
Model 2	1.00	1.02 (0.88, 1.19)	1.11 (0.97, 1.26)	1.09 (0.95, 1.25)	1.01 (0.88, 1.16)	0.55
Community 3 “Sweets and snacks”						
Number of participants	19,872	19,873	19,872	19,873	19,872	
Number of cases	439	436	409	349	245	
Model 1	1.00	1.03 (0.90, 1.18)	1.11 (0.96, 1.27)	1.18 (1.02, 1.37)	1.26 (1.06, 1.50)	0.003
Model 2	1.00	1.05 (0.92, 1.21)	1.16 (1.01, 1.34)	1.25 (1.08, 1.46)	1.34 (1.12, 1.59)	0.0001
Community 4 “Plant-based”						
Number of participants	19,872	19,874	19,871	19,873	19,872	
Number of cases	347	370	378	433	350	
Model 1	1.00	1.07 (0.92, 1.24)	0.97 (0.84, 1.12)	1.07 (0.92, 1.23)	0.91 (0.78, 1.06)	0.26
Model 2	1.00	1.05 (0.91, 1.22)	0.96 (0.82, 1.11)	1.07 (0.93, 1.24)	0.96 (0.82, 1.12)	0.77
Community 5 “Healthy”						
Number of participants	19,872	19,873	19,872	19,873	19,872	
Number of cases	225	382	392	429	450	
Model 1	1.00	1.05 (0.89, 1.24)	0.86 (0.73, 1.02)	0.84 (0.71, 0.98)	0.83 (0.70, 0.97)	0.0003
Model 2	1.00	1.11 (0.94, 1.32)	0.95 (0.81, 1.13)	0.96 (0.81, 1.13)	0.99 (0.83, 1.17)	0.27

Model 1: Adjusted for energy (in kilocalories per day), calculated from the mean of all available 24-h dietary records at baseline.

Model 2: Adjusted for energy, sex, household monthly income, education level, marital status, employment status, smoking status, number of cigarettes packs per year, physical activity level, body mass index, family history of cardiovascular disease, and number of dietary records.

In sensitivity analyses, the high hazards of CVD associated with higher scores of the dietary pattern community 3 “Sweets and snacks” remained significant when further adjusted for the PNNS 2 score (HR_Q5 vs Q1_: 1.32; 95% CI: 1.11, 1.57; *P*-trend = 0.0002) but not after adjustment for the %UPF (HR_Q5 vs Q1_ : 1.19; 95% CI: 0.98, 1.43; Supplemental Table 7), although the linear trend remained significant (*P*-trend = 0.02). Implementing cubic spline transformation for the dietary pattern network scores in the Cox proportional hazard models confirmed the significant and linear overall association between community 3 “Sweets and snacks” scores and CVD incidence (data not shown). Finally, the associations between dietary pattern network scores and incident CVD were examined while considering hard CVD events only (*n* = 98,061) and after the exclusion of participants with prevalent type 2 diabetes, hypertension, and dyslipidemia (*n* = 85,049). The results were consistent with the primary findings, confirming that community 3 “Sweets and snacks” network scores were significantly associated with CVD incidence. However, the associations were generally stronger than those reported in the primary results (Supplemental Tables 8 and 9).

## Discussion

The application of data-based approaches to study dietary patterns can assist in elucidating the intricate interrelationships between foods and health outcomes. Conventional data-based approaches, such as principal component analysis and reduced rank regression, are subject to certain limitations, which may result in the incomplete characterization of the structure and relationships among food groups. The fields of data science and machine learning are evolving rapidly, leading to the development of innovative approaches, such as GGMs and the Louvain algorithm, which may provide novel perspectives on the complex structures of dietary patterns and their association with clinical outcomes. The objective of this exploratory study was 2-fold: first, to identify dietary pattern networks using a combination of GGMs and the Louvain algorithm; and second, to examine the associations between the identified dietary pattern networks and CVD incidence in a sample of the French population.

Five distinct dietary pattern networks were identified, corresponding to each community detected by the Louvain algorithm in the GGM-generated network. Networks reflected healthier dietary patterns (community 4 “Plant-based” and community 5 “Healthy”) or a less healthy dietary pattern (community 3 “Sweets and snacks”). This method also identified dietary pattern networks that were not associated with diet quality but rather reflected typical food intake patterns observed in this sample of French participants (community 1 “Appetizers” and community 2 “Breakfast”). In accordance with our findings, numerous studies have demonstrated that GGMs represent an adequate exploratory method to study dietary patterns [23,28] and to identify healthy/less healthy or intrinsic dietary pattern networks within a population [23,27]. It is our contention that novel approaches such as combining GGMs and the Louvain algorithm can contribute to a more nuanced understanding of the intricate and significant structures of food groups representing dietary patterns in a population. Although healthy dietary patterns have been extensively documented, this approach revealed dietary patterns that are highly intrinsic to the French population, allowing understanding of how these patterns may be related to health outcomes. Although further validation is warranted through replication in similar populations, for example, these dietary patterns could ultimately contribute to informing future public health policies and interventions.

One of the 5 dietary pattern networks demonstrated a positive association with the incidence of CVD. Indeed, the community 3 “Sweets and snacks” scores, which reflected the global consumption of ultraprocessed sweets and snacks, demonstrated a linear association with a higher CVD incidence, independent of energy intake, potential confounders, diet quality, and other dietary pattern network scores. Indeed, the HR of CVD of participants in the top fifth compared with those in the bottom fifth of community 3 “Sweets and snacks” score distribution was ≤34% higher. This association was amplified when considering only hard CVD events (≤50% higher HR of CVD), after exclusion of participants with prevalent nutrition-related diseases (≤51% HR of CVD), which remained statistically significant even after adjusting for the %UPF in the diet. This suggests that the dietary pattern itself, which reflects food groups often consumed together in this cohort of adults from France, is inherently linked to the incidence of CVD, irrespective of various indices of diet quality and numerous other CVD risk factors. The community 5 “Healthy” dietary pattern comprised foods frequently recommended in dietary guidelines, and as anticipated, this pattern was associated with a lower incidence of CVD. However, this association was no longer significant after adjustment for important covariates. Community 1 “Appetizers,” which included appetizer foods such as charcuteries, cheese, and alcoholic beverages, was associated with higher CVD incidence, but this association was no longer significant after further adjustment for confounders. The dietary pattern scores for community 4 “Plant-based” and community 2 “Breakfast” were not associated with CVD incidence. These findings indicate that the community 3 “Sweets and snacks” reflects the dietary pattern network most closely associated with cardiovascular health in this population. Although 5 data-driven dietary pattern scores were tested, the significant association between the community 3 “Sweets and snacks” pattern and CVD risk remained below a conservative Bonferroni threshold (*P* < 0.01) and was consistently observed across several sensitivity analyses, including adjustment for diet quality, exclusion of high-risk participants, and nonlinear modeling. Nonetheless, we acknowledge that the possibility of a false-positive finding cannot be entirely excluded.

Previous studies have used more traditional approaches, such as principal component analysis or reduced rank regression, to identify empirically derived dietary patterns and to test associations with CVD incidence. To our knowledge, no such study has used GGMs and the Louvain algorithm for such analyses. The Louvain algorithm compared with traditional approaches like principal component analysis or reduced rank regression is fundamentally different in terms of assumptions and objectives [46]. Consequently, it is challenging to make direct comparisons between studies that have used different methodologies to identify dietary patterns. Nevertheless, our findings are consistent with those of other studies, which have demonstrated that dietary patterns characterized by UPFs high in fat and sugar, such as those found in the community 3 “Sweets and snacks” pattern, are associated with a higher incidence of CVD [38,47,48]. The dietary pattern network scores generated by the GGMs and Louvain algorithm were determined by 2 factors: the amounts of foods consumed within each food group and the associations among all food groups. This approach may therefore assist in the identification of dietary patterns comprising foods that are frequently consumed, often together, and that should be more prominently targeted for the prevention of CVD in a particular population. As GGMs and the Louvain algorithm are relatively novel approaches to characterize dietary patterns, these findings must be validated in other large-scale observational prospective studies with different population characteristics. It is of paramount importance to note that the dietary patterns identified through the data-driven GGM method in conjunction with the Louvain algorithm are population-specific, and thus, results cannot be extrapolated to other populations.

It is important to note the limitations of the present study. First, although previous studies have shown that GGMs are robust to dietary intake variables that are not perfectly normally distributed [23], it is possible that GGMs may perform best with Gaussian-distributed data. This is rarely the case for dietary intake data, even when a transformation is applied to dietary intake variables. In the present study, all variables were not perfectly normally distributed after log transformation. Second, the NutriNet-Santé cohort is not representative of the French population. Thus, the dietary pattern networks identified cannot be generalized to the entire French population. Third, eigenvector centrality and grams of food groups consumed were employed as variables to generate dietary pattern network scores, as previously described by Hoang et al. [28]. Exploring other centrality measures in future research is warranted. Finally, residual confounding cannot be excluded and causation cannot be inferred because of the observational nature of the study.

The study also possesses a number of notable strengths. First, a population-based cohort with a large sample size was utilized for the analyses. Second, a minimum of 2 24-h dietary records validated in the French population [34,35] was used to measure dietary intakes in the sample. Third, the majority of studies that have employed GGMs have not utilized the Louvain algorithm for community detection. The utilization of the Louvain algorithm in conjunction with GGMs permitted the identification of latent structures within dietary intake data, which are not automatically generated through the use of GGMs. Fourth, unlike conventional methods such as principal component analysis and reduced rank regression, GGM identifies conditional dependencies between pairs of food groups by adjusting for the effects of other food groups, thereby accounting for confounding between dietary intake variables. Finally, to our knowledge, this is the first study to use the GGM–Louvain method to identify dietary pattern networks and to study associations with CVD incidence in a French population.

In conclusion, GGMs and the Louvain algorithm are innovative empirical approaches for deriving dietary pattern networks that provide insights into the most prevalent dietary patterns in a population and the relationships between food groups and associations with clinical outcomes. The results indicate the existence of a dietary pattern network characterized by the consumption of sweets and snacks, which is associated with CVD incidence, independent of the effects of multiple confounders and other diet-related covariates, including overall diet quality, in a sample of the French population. Such findings may provide a framework for identifying the food groups that should be prioritized in public health strategies aimed at reducing the diet-related burden of CVD and other chronic diseases. Future intervention studies could explore whether targeting highly central communities within a population’s dietary network, such as the “Sweets and snacks” community in the NutriNet-Santé cohort, could influence broader eating behaviors through downstream effects on the consumption of other co-occurring food groups. This network-based approach could also lead to the design of novel behavioral intervention strategies, whereby modifying a few key nodes (foods) in the dietary network might shift the entire pattern toward healthier choices. In addition, future research could explore how dietary networks evolve over time and how they are related to underlying biological mechanisms. Such analyses may provide greater mechanistic insight into how specific dietary structures influence chronic disease development. Further studies are required to investigate how the use of other machine learning algorithms can provide insightful information on the association between dietary pattern and risk of clinical outcomes in large-scale population-based studies.

## Author contributions

The authors’ responsibilities were as follows – MC, BL, LKF, JMH, MT: designed the study; MC: performed statistical analyses and drafted the manuscript; BL, LKF: supervised the statistical analyses and the writing; LKF: primary responsibility for the final content and is the guarantor; and all authors: contributed to the data interpretation and revised each draft for important intellectual content, and read and approved the final manuscript.

## Data availability

Data described in the manuscript, code book, and analytic code will be made available upon request pending application and approval.

## Funding

The NutriNet-Santé study was supported by the Ministère de la Santé, Santé Publique France, Institut National de la Santé et de la Recherche Médicale (INSERM), Institut National de la Recherche Agronomique (INRA), Conservatoire National des Arts et Métiers (CNAM), and Université Paris 13. BS was funded by the French National Cancer Institute (grant No INCa_8085) and Fondation de France. Researchers were independent from funders. Funders had no role in the study design, the collection, analysis, and interpretation of data, the writing of the report, and the decision to submit the article for publication.

## Conflict of interest

The authors report no conflicts of interest.
