# Supplementary material for: Associations between Dietary Pattern Networks Derived from Machine Learning Algorithms and Cardiovascular Disease Risk in the NutriNet-Santé Cohort
Source: J Nutr. 2025 Sep 23;155(11):4033–42. doi: 10.1016/j.tjnut.2025.09.014 (PMC12799445; doi:10.1016/j.tjnut.2025.09.014)

| **Supplementary Table 1**. Eigenvector centrality values of food groups included in the dietary pattern network scores. | | |
| --- | --- | --- |
| **Food group** | | **Eigenvector centrality** |
| **Community 1** | |  |
|  | Bread and crackers | 0.0533 |
|  | Charcuteries | 0.0522 |
|  | Cheese | 0.0390 |
|  | Meats | 0.0390 |
|  | Alcoholic beverages | 0.0329 |
|  | Butter | 0.0314 |
|  | Sauces and salad dressings | 0.0269 |
|  | Appetizer products | 0.0182 |
|  | Starchy foods and tubers | 0.0154 |
|  | Other fats | 0.0124 |
|  | Eggs | 0.0014 |
| **Community 2** | |  |
|  | Breakfast cereals and soft bars | 0.0195 |
|  | Milk | 0.0174 |
| **Community 3** | |  |
|  | Cakes | 0.0191 |
|  | Non-alcoholic sugary beverages | 0.0380 |
|  | Chocolate and sweets | 0.0268 |
|  | Pastries | 0.0260 |
|  | Dairy-based desserts | 0.0202 |
|  | Fast food and snacks | 0.0169 |
|  | Cookies | 0.0074 |
|  | Fruits juices 100% pure | 0.0073 |
| **Community 4** | |  |
|  | Dairy substitutes | 0.0685 |
|  | Protein substitutes | 0.0495 |
|  | Nuts | 0.0444 |
|  | Whole-grain cereal products | 0.0425 |
|  | Legumes | 0.0313 |
|  | Other cereal products | 0.0221 |
|  | Plant-based charcuteries | 0.0135 |
|  | Plant-based dairy desserts | 0.0135 |
|  | Pasta | 0.0054 |
| **Community 5** | |  |
|  | Fish and seafood | 0.0152 |
|  | Vegetables | 0.0469 |
|  | Whole-grain breads and crackers | 0.0388 |
|  | Fruits | 0.0261 |
|  | Yoghurts | 0.0255 |
|  | Non-alcoholic and non-sugary beverages | 0.0200 |
|  | Pork and poultry hams | 0.0086 |
|  | Poultry | 0.0057 |
|  | Rice | 0.0017 |
| *Groups not included in dietary pattern network scores: Fish charcuteries, offals, vegetable juices. | | |

| **Supplementary Table 2**. Median consumption of food groups and diet quality indices in the sample selected from the NutriNet-Santé cohort (n = 99 362) | |
| --- | --- |
| **Food group, g/day** | **Median (Q1-Q3)** |
| Fruits | 155.7 (75.8-255.1) |
| Vegetables | 220.5 (142.7-317.9) |
| Fish and seafood | 27.7 (4.8-54.5) |
| Fish and seafood charcuteries | 0.0 (0.0-0.0) |
| Meats | 32.1 (8.0-58.6) |
| Charcuteries | 11. (0.0-27.5) |
| Poultry | 15.9 (0.0-37.1) |
| Pork and poultry hams | 0.0 (0.0-14.3) |
| Offals | 0.0 (0.0-0.0) |
| Protein substitutes | 0.0 (0.0-0.0) |
| Plant-based charcuteries | 0.0 (0.0-0.0) |
| Legumes | 0.0 (0.0-12.5) |
| Nuts | 0.0 (0.0-5.4) |
| Eggs | 4.3 (0.0-17.9) |
| Milk | 12.6 (0.0-123.2) |
| Cheese | 27.3 (12.6-45.5) |
| Yoghurt | 55.4 (0.0-122.6) |
| Dairy substitutes | 0.0 (0.0-0.0) |
| Dairy-based desserts | 17.9 (0.0-50.0) |
| Plant-based dairy desserts | 0.0 (0.0-0.0) |
| Starchy foods and tubers | 32.1 (5.7-63.6) |
| Breads and crackers | 57.1 (25.7-98.6) |
| Whole-grain breads and crackers | 11.4 (0.0-39.1) |
| Pasta | 23.8 (0.0-53.6) |
| Rice | 5.7 (0.0-30.8) |
| Other cereal products | 0.0 (0.0-17.9) |
| Whole-grain cereal products | 0.0 (0.0-0.0) |
| Cakes | 26.8 (0.0-58.9) |
| Cookies | 0.0 (0.0-10.7) |
| Pastries | 0.0 (0.0-13.9) |
| Breakfast cereals and soft bars | 0.0 (0.0-10.7) |
| Chocolate, candies and sweets | 39.1 (19.0-66.0) |
| Non-alcoholic sugary beverages | 0.0 (0.0-51.8) |
| Non-alcoholic and non-sugary beverages | 1016.8 (714.3-1381.8) |
| Alcoholic beverages | 42.9 (0.0-132.3) |
| Fruit juices 100% pure | 12.9 (0.0-91.6) |
| Vegetable juices | 0.0 (0.0-0.0) |
| Sauces and salad dressings | 13.2 (5.7-23.7) |
| Butter | 3.9 (0.0-10.0) |
| Other fats | 11.1 (5.4-19.3) |
| Fast food and snacks | 19.5 (0.0-55.4) |
| Appetizer products | 0.0 (0.0-4.3) |

| **Supplementary Table 3.** Precision matrix presenting the partial pairwise correlations between food groups adjusted for the effects of all other food groups in the sample. | | | | | | |
| --- | --- | --- | --- | --- | --- | --- |
|  | **Fruits** | **Charcuteries** | **Vegetables** | **Fish and seafood** | **Fish charcuteries** | **Plant-based charcuteries** |
| **Fruits** | 1.091731 | 0 | -0.25146 | -0.04937 | 0 | 0 |
| **Charcuteries** | 0 | 1.029617 | 0 | 0 | 0 | 0 |
| **Vegetables** | -0.25146 | 0 | 1.118945 | -0.04252 | 0 | 0 |
| **Fish and seafood** | -0.04937 | 0 | -0.04252 | 1.010788 | 0 | 0 |
| **Fish charcuteries** | 0 | 0 | 0 | 0 | 1 | 0 |
| **Plant-based charcuteries** | 0 | 0 | 0 | 0 | 0 | 1.03484 |
| **Pork and poultry hams** | 0 | 0 | 0 | 0 | 0 | 0 |
| **Meats** | 0 | -0.06738 | 0 | 0 | 0 | 0 |
| **Poultry** | 0 | 0 | 0 | 0 | 0 | 0 |
| **Offals** | 0 | 0 | 0 | 0 | 0 | 0 |
| **Protein substitutes** | 0 | 0.053369 | 0 | 0 | 0 | -0.16985 |
| **Eggs** | 0 | 0 | 0 | 0 | 0 | 0 |
| **Milk** | 0 | 0 | 0 | 0 | 0 | 0 |
| **Dairy substitutes** | 0 | 0.026148 | -0.02504 | 0 | 0 | -0.05488 |
| **Cheese** | 0 | -0.03872 | 0 | 0 | 0 | 0 |
| **Yoghurts** | -0.06317 | 0 | -0.06111 | -0.0329 | 0 | 0 |
| **Dairy-based desserts** | 0 | 0 | 0 | 0 | 0 | 0 |
| **Plant-based dairy desserts** | 0 | 0 | 0 | 0 | 0 | 0 |
| **Starchy foods and tubers** | 0 | -0.02035 | 0 | 0 | 0 | 0 |
| **Bread and crackers** | 0 | -0.07702 | 0 | 0 | 0 | 0 |
| **Whole-grain breads and crackers** | -0.07717 | 0 | -0.09464 | 0 | 0 | 0 |

| **Supplementary Table 3 (cont).** Precision matrix presenting the partial pairwise correlations between food groups adjusted for the effects of all other food groups in the sample. | | | | | | |
| --- | --- | --- | --- | --- | --- | --- |
|  | **Fruits** | **Charcuteries** | **Vegetables** | **Fish and seafood** | **Fish charcuteries** | **Plant-based charcuteries** |
| **Breakfast cereals and soft bars** | 0 | 0 | 0 | 0 | 0 | 0 |
| **Pasta** | 0 | 0 | 0.03411 | 0 | 0 | 0 |
| **Rice** | 0 | 0 | 0 | -0.05726 | 0 | 0 |
| **Other cereal products** | 0 | 0 | 0 | 0 | 0 | 0 |
| **Whole-grain cereal products** | 0 | 0 | -0.01013 | 0 | 0 | 0 |
| **Cookies** | 0 | 0 | 0 | 0 | 0 | 0 |
| **Pastries** | 0 | -0.00824 | 0.028456 | 0 | 0 | 0 |
| **Non-alcoholic sugary beverages** | 0.027413 | -0.002 | 0.091696 | 0 | 0 | 0 |
| **Non-alcoholic and non-sugary beverages** | -0.06085 | 0 | -0.08091 | 0 | 0 | 0 |
| **Fruit juices 100% pure** | 0 | 0 | 0 | 0 | 0 | 0 |
| **Vegetable juices** | 0 | 0 | 0 | 0 | 0 | 0 |
| **Alcoholic beverages** | 0 | -0.09016 | 0 | -0.02579 | 0 | 0 |
| **Sauces and salad dressings** | 0 | -0.00906 | 0 | 0 | 0 | 0 |
| **Butter** | 0 | -0.0055 | 0 | 0 | 0 | 0 |
| **Other fats** | 0 | 0 | -0.0309 | 0 | 0 | 0 |
| **Chocolate and sweets** | 0 | 0 | 0 | 0 | 0 | 0 |
| **Nuts** | -0.05473 | 0 | -0.05438 | 0 | 0 | 0 |
| **Fast food and snacks** | 0 | 0 | 0.092614 | 0 | 0 | 0 |
| **Appetizer products** | 0 | -0.02263 | 0 | 0 | 0 | 0 |
| **Cakes** | 0 | -0.00864 | 0 | 0 | 0 | 0 |
| **Legumes** | 0 | 0 | -0.00812 | 0 | 0 | 0 |

| **Supplementary Table 3 (cont).** Precision matrix presenting the partial pairwise correlations between food groups adjusted for the effects of all other food groups in the sample. | | | | | | |
| --- | --- | --- | --- | --- | --- | --- |
|  | **Pork and poultry hams** | **Meats** | **Poultry** | **Offals** | **Protein substitutes** | **Eggs** |
| **Fruits** | 0 | 0 | 0 | 0 | 0 | 0 |
| **Charcuteries** | 0 | -0.06738 | 0 | 0 | 0.053369 | 0 |
| **Vegetables** | 0 | 0 | 0 | 0 | 0 | 0 |
| **Fish and seafood** | 0 | 0 | 0 | 0 | 0 | 0 |
| **Fish charcuteries** | 0 | 0 | 0 | 0 | 0 | 0 |
| **Plant-based charcuteries** | 0 | 0 | 0 | 0 | -0.16985 | 0 |
| **Pork and poultry hams** | 1.000088 | 0 | 0 | 0 | 0.006826 | 0 |
| **Meats** | 0 | 1.026857 | 0 | 0 | 0.098921 | 0 |
| **Poultry** | 0 | 0 | 1.000808 | 0 | 0.028445 | 0 |
| **Offals** | 0 | 0 | 0 | 1 | 0 | 0 |
| **Protein substitutes** | 0.006826 | 0.098921 | 0.028445 | 0 | 1.104101 | 0 |
| **Eggs** | 0 | 0 | 0 | 0 | 0 | 1.000767 |
| **Milk** | 0 | 0 | 0 | 0 | 0 | 0 |
| **Dairy substitutes** | 0 | 0.055849 | 0 | 0 | -0.20324 | 0 |
| **Cheese** | 0 | 0 | 0 | 0 | 0.001355 | 0 |
| **Yoghurts** | -0.00638 | 0 | 0 | 0 | 0 | 0 |
| **Dairy-based desserts** | 0 | 0 | 0 | 0 | 0 | 0 |
| **Plant-based dairy desserts** | 0 | 0 | 0 | 0 | -0.02623 | 0 |
| **Starchy foods and tubers** | 0 | -0.05184 | 0 | 0 | 0 | 0 |
| **Bread and crackers** | 0 | -0.03421 | 0 | 0 | 0.006657 | 0 |
| **Whole-grain breads and crackers** | 0 | 0 | 0 | 0 | 0 | 0 |
| **Supplementary Table 3 (cont).** Precision matrix presenting the partial pairwise correlations between food groups adjusted for the effects of all other food groups in the sample. | | | | | | |
|  | **Pork and poultry hams** | **Meats** | **Poultry** | **Offals** | **Protein substitutes** | **Eggs** |
| **Breakfast cereals and soft bars** | 0 | 0 | 0 | 0 | 0 | 0 |
| **Pasta** | 0 | 0 | 0 | 0 | 0 | 0 |
| **Rice** | 0 | 0 | 0 | 0 | 0 | 0 |
| **Other cereal products** | 0 | 0 | 0 | 0 | -0.00436 | 0 |
| **Whole-grain cereal products** | 0 | 0.004942 | 0 | 0 | -0.07201 | 0 |
| **Cookies** | 0 | 0 | 0 | 0 | 0 | 0 |
| **Pastries** | 0 | 0 | 0 | 0 | 0 | 0 |
| **Non-alcoholic sugary beverages** | 0 | 0 | 0 | 0 | 0 | 0 |
| **Non-alcoholic and non-sugary beverages** | 0 | 0 | 0 | 0 | 0 | 0 |
| **Fruit juices 100% pure** | 0 | 0 | 0 | 0 | 0 | 0 |
| **Vegetable juices** | 0 | 0 | 0 | 0 | 0 | 0 |
| **Alcoholic beverages** | 0 | -0.01442 | 0 | 0 | 0 | 0 |
| **Sauces and salad dressings** | 0 | -0.02383 | 0 | 0 | 0 | 0 |
| **Butter** | 0 | 0 | 0 | 0 | 0 | 0 |
| **Other fats** | 0 | 0 | 0 | 0 | 0 | -0.0277 |
| **Chocolate and sweets** | 0 | 0 | 0 | 0 | 0 | 0 |
| **Nuts** | 0 | 0 | 0 | 0 | -0.0452 | 0 |
| **Fast food and snacks** | 0 | 0 | 0 | 0 | 0 | 0 |
| **Appetizer products** | 0 | 0 | 0 | 0 | 0 | 0 |
| **Cakes** | 0 | 0 | 0 | 0 | 0 | 0 |
| **Legumes** | 0 | 0 | 0 | 0 | -0.0209 | 0 |
| **Supplementary Table 3 (cont).** Precision matrix presenting the partial pairwise correlations between food groups adjusted for the effects of all other food groups in the sample. | | | | | | |
|  | **Milk** | **Dairy substitutes** | **Cheese** | **Yoghurts** | **Dairy-based desserts** | **Plant-based dairy desserts** |
| **Fruits** | 0 | 0 | 0 | -0.06317 | 0 | 0 |
| **Charcuteries** | 0 | 0.026148 | -0.03872 | 0 | 0 | 0 |
| **Vegetables** | 0 | -0.02504 | 0 | -0.06111 | 0 | 0 |
| **Fish and seafood** | 0 | 0 | 0 | -0.0329 | 0 | 0 |
| **Fish charcuteries** | 0 | 0 | 0 | 0 | 0 | 0 |
| **Plant-based charcuteries** | 0 | -0.05488 | 0 | 0 | 0 | 0 |
| **Pork and poultry hams** | 0 | 0 | 0 | -0.00638 | 0 | 0 |
| **Meats** | 0 | 0.055849 | 0 | 0 | 0 | 0 |
| **Poultry** | 0 | 0 | 0 | 0 | 0 | 0 |
| **Offals** | 0 | 0 | 0 | 0 | 0 | 0 |
| **Protein substitutes** | 0 | -0.20324 | 0.001355 | 0 | 0 | -0.02623 |
| **Eggs** | 0 | 0 | 0 | 0 | 0 | 0 |
| **Milk** | 1.010966 | 0.067469 | 0 | 0 | -0.00069 | 0 |
| **Dairy substitutes** | 0.067469 | 1.126334 | 0.006828 | 0.027638 | 0.010727 | -0.15527 |
| **Cheese** | 0 | 0.006828 | 1.020112 | 0 | 0 | 0 |
| **Yoghurts** | 0 | 0.027638 | 0 | 1.012659 | 0 | 0 |
| **Dairy-based desserts** | -0.00069 | 0.010727 | 0 | 0 | 1.000698 | 0 |
| **Plant-based dairy desserts** | 0 | -0.15527 | 0 | 0 | 0 | 1.025955 |
| **Starchy foods and tubers** | 0 | 0 | 0 | 0 | 0 | 0 |
| **Bread and crackers** | 0 | 0.040873 | -0.09642 | 0 | 0 | 0 |
| **Whole-grain breads and crackers** | 0 | -0.00275 | 0 | 0 | 0 | 0 |
| **Supplementary Table 3 (cont).** Precision matrix presenting the partial pairwise correlations between food groups adjusted for the effects of all other food groups in the sample. | | | | | | |
|  | **Milk** | **Dairy substitutes** | **Cheese** | **Yoghurts** | **Dairy-based desserts** | **Plant-based dairy desserts** |
| **Breakfast cereals and soft bars** | -0.08848 | -0.11793 | 0 | 0 | 0 | 0 |
| **Pasta** | 0 | 0 | 0 | 0 | 0 | 0 |
| **Rice** | 0 | 0 | 0 | 0 | 0 | 0 |
| **Other cereal products** | 0 | -0.01327 | 0 | 0 | 0 | 0 |
| **Whole-grain cereal products** | 0 | -0.0858 | 0 | 0 | 0 | 0 |
| **Cookies** | 0 | 0 | 0 | 0 | 0 | 0 |
| **Pastries** | 0 | 0 | 0 | 0 | -0.01915 | 0 |
| **Non-alcoholic sugary beverages** | 0 | 0 | 0 | 0.004865 | -0.0057 | 0 |
| **Non-alcoholic and non-sugary beverages** | 0 | 0 | 0 | -0.01848 | 0 | 0 |
| **Fruit juices 100% pure** | 0 | 0 | 0 | 0 | 0 | 0 |
| **Vegetable juices** | 0 | 0 | 0 | 0 | 0 | 0 |
| **Alcoholic beverages** | 0 | 0 | -0.05664 | 0 | 0 | 0 |
| **Sauces and salad dressings** | 0 | 0 | -0.00628 | 0 | 0 | 0 |
| **Butter** | 0 | 0.012138 | -0.05687 | 0 | 0 | 0 |
| **Other fats** | 0 | 0 | 0 | 0 | 0 | 0 |
| **Chocolate and sweets** | 0 | 0 | -0.00224 | 0 | -0.01263 | 0 |
| **Nuts** | 0.00328 | -0.10735 | 0 | 0 | 0 | 0 |
| **Fast food and snacks** | 0 | 0 | 0 | 0 | 0 | 0 |
| **Appetizer products** | 0 | 0 | 0 | 0 | 0 | 0 |
| **Cakes** | 0 | 0 | 0 | 0 | 0 | 0 |
| **Legumes** | 0 | -0.02281 | 0 | 0 | 0 | 0 |

| **Supplementary Table 3 (cont).** Precision matrix presenting the partial pairwise correlations between food groups adjusted for the effects of all other food groups in the sample. | | | | | | |
| --- | --- | --- | --- | --- | --- | --- |
|  | **Starchy foods and tubers** | **Bread and crackers** | **Whole-grain breads and crackers** | **Breakfast cereals and soft bars** | **Pasta** | **Rice** |
| **Fruits** | 0 | 0 | -0.07717 | 0 | 0 | 0 |
| **Charcuteries** | -0.02035 | -0.07702 | 0 | 0 | 0 | 0 |
| **Vegetables** | 0 | 0 | -0.09464 | 0 | 0.03411 | 0 |
| **Fish and seafood** | 0 | 0 | 0 | 0 | 0 | -0.05726 |
| **Fish charcuteries** | 0 | 0 | 0 | 0 | 0 | 0 |
| **Plant-based charcuteries** | 0 | 0 | 0 | 0 | 0 | 0 |
| **Pork and poultry hams** | 0 | 0 | 0 | 0 | 0 | 0 |
| **Meats** | -0.05184 | -0.03421 | 0 | 0 | 0 | 0 |
| **Poultry** | 0 | 0 | 0 | 0 | 0 | 0 |
| **Offals** | 0 | 0 | 0 | 0 | 0 | 0 |
| **Protein substitutes** | 0 | 0.006657 | 0 | 0 | 0 | 0 |
| **Eggs** | 0 | 0 | 0 | 0 | 0 | 0 |
| **Milk** | 0 | 0 | 0 | -0.08848 | 0 | 0 |
| **Dairy substitutes** | 0 | 0.040873 | -0.00275 | -0.11793 | 0 | 0 |
| **Cheese** | 0 | -0.09642 | 0 | 0 | 0 | 0 |
| **Yoghurts** | 0 | 0 | 0 | 0 | 0 | 0 |
| **Dairy-based desserts** | 0 | 0 | 0 | 0 | 0 | 0 |
| **Plant-based dairy desserts** | 0 | 0 | 0 | 0 | 0 | 0 |
| **Starchy foods and tubers** | 1.012002 | 0 | 0 | 0 | 0 | 0 |
| **Bread and crackers** | 0 | 1.082606 | 0.190277 | 0.034917 | 0 | 0 |
| **Whole-grain breads and crackers** | 0 | 0.190277 | 1.062635 | 0 | 0 | 0 |

| **Supplementary Table 3 (cont).** Precision matrix presenting the partial pairwise correlations between food groups adjusted for the effects of all other food groups in the sample. | | | | | | |
| --- | --- | --- | --- | --- | --- | --- |
|  | **Starchy foods and tubers** | **Bread and crackers** | **Whole-grain breads and crackers** | **Breakfast cereals and soft bars** | **Pasta** | **Rice** |
| **Breakfast cereals and soft bars** | 0 | 0.034917 | 0 | 1.023623 | 0 | 0 |
| **Pasta** | 0 | 0 | 0 | 0 | 1.001162 | 0 |
| **Rice** | 0 | 0 | 0 | 0 | 0 | 1.003268 |
| **Other cereal products** | 0 | 0 | 0 | 0 | 0 | 0 |
| **Whole-grain cereal products** | 0 | 0.022755 | -0.07006 | 0 | 0 | 0 |
| **Cookies** | 0 | 0 | 0 | 0 | 0 | 0 |
| **Pastries** | 0 | 0 | 0 | 0 | 0 | 0 |
| **Non-alcoholic sugary beverages** | 0 | 0 | 0.001418 | 0 | 0 | 0 |
| **Non-alcoholic and non-sugary beverages** | 0 | 0 | -0.01907 | 0 | 0 | 0 |
| **Fruit juices 100% pure** | 0 | 0 | 0 | 0 | 0 | 0 |
| **Vegetable juices** | 0 | 0 | 0 | 0 | 0 | 0 |
| **Alcoholic beverages** | 0 | -7.4E-05 | 0 | 0 | 0 | 0 |
| **Sauces and salad dressings** | 0 | -0.01263 | 0 | 0 | 0 | 0 |
| **Butter** | -0.01944 | -0.13888 | 0 | 0.036301 | 0 | 0 |
| **Other fats** | -0.09166 | 0 | 0 | 0 | 0 | 0 |
| **Chocolate and sweets** | 0 | -0.05047 | 0 | 0 | 0 | 0 |
| **Nuts** | 0 | 0 | -0.03214 | 0 | 0 | 0 |
| **Fast food and snacks** | 0 | 0 | 0 | 0 | 0 | 0 |
| **Appetizer products** | 0 | 0 | 0 | 0 | 0 | 0 |
| **Cakes** | 0 | 0 | 0 | 0 | 0 | 0 |
| **Legumes** | 0 | 0 | 0 | 0 | 0 | 0 |

| **Supplementary Table 3 (cont).** Precision matrix presenting the partial pairwise correlations between food groups adjusted for the effects of all other food groups in the sample. | | | | | | |
| --- | --- | --- | --- | --- | --- | --- |
|  | **Other cereal products** | **Whole-grain cereal products** | **Cookies** | **Pastries** | **Non-alcoholic sugary beverages** | **Non-alcoholic and non-sugary beverages** |
| **Fruits** | 0 | 0 | 0 | 0 | 0.027413 | -0.06085 |
| **Charcuteries** | 0 | 0 | 0 | -0.00824 | -0.002 | 0 |
| **Vegetables** | 0 | -0.01013 | 0 | 0.028456 | 0.091696 | -0.08091 |
| **Fish and seafood** | 0 | 0 | 0 | 0 | 0 | 0 |
| **Fish charcuteries** | 0 | 0 | 0 | 0 | 0 | 0 |
| **Plant-based charcuteries** | 0 | 0 | 0 | 0 | 0 | 0 |
| **Pork and poultry hams** | 0 | 0 | 0 | 0 | 0 | 0 |
| **Meats** | 0 | 0.004942 | 0 | 0 | 0 | 0 |
| **Poultry** | 0 | 0 | 0 | 0 | 0 | 0 |
| **Offals** | 0 | 0 | 0 | 0 | 0 | 0 |
| **Protein substitutes** | -0.00436 | -0.07201 | 0 | 0 | 0 | 0 |
| **Eggs** | 0 | 0 | 0 | 0 | 0 | 0 |
| **Milk** | 0 | 0 | 0 | 0 | 0 | 0 |
| **Dairy substitutes** | -0.01327 | -0.0858 | 0 | 0 | 0 | 0 |
| **Cheese** | 0 | 0 | 0 | 0 | 0 | 0 |
| **Yoghurts** | 0 | 0 | 0 | 0 | 0.004865 | -0.01848 |
| **Dairy-based desserts** | 0 | 0 | 0 | -0.01915 | -0.0057 | 0 |
| **Plant-based dairy desserts** | 0 | 0 | 0 | 0 | 0 | 0 |
| **Starchy foods and tubers** | 0 | 0 | 0 | 0 | 0 | 0 |
| **Bread and crackers** | 0 | 0.022755 | 0 | 0 | 0 | 0 |
| **Whole-grain breads and crackers** | 0 | -0.07006 | 0 | 0 | 0.001418 | -0.01907 |

| **Supplementary Table 3 (cont).** Precision matrix presenting the partial pairwise correlations between food groups adjusted for the effects of all other food groups in the sample. | | | | | | |
| --- | --- | --- | --- | --- | --- | --- |
|  | **Other cereal products** | **Whole-grain cereal products** | **Cookies** | **Pastries** | **Non-alcoholic sugary beverages** | **Non-alcoholic and non-sugary beverages** |
| **Breakfast cereals and soft bars** | 0 | 0 | 0 | 0 | 0 | 0 |
| **Pasta** | 0 | 0 | 0 | 0 | 0 | 0 |
| **Rice** | 0 | 0 | 0 | 0 | 0 | 0 |
| **Other cereal products** | 1.000698 | 0 | 0 | 0 | 0 | 0 |
| **Whole-grain cereal products** | 0 | 1.026254 | 0 | 0 | 0 | 0 |
| **Cookies** | 0 | 0 | 1.001369 | 0 | -0.00356 | 0 |
| **Pastries** | 0 | 0 | 0 | 1.006318 | -0.05619 | 0 |
| **Non-alcoholic sugary beverages** | 0 | 0 | -0.00356 | -0.05619 | 1.020922 | 0.010958 |
| **Non-alcoholic and non-sugary beverages** | 0 | 0 | 0 | 0 | 0.010958 | 1.014708 |
| **Fruit juices 100% pure** | 0 | 0 | 0 | -0.00327 | -0.01046 | 0 |
| **Vegetable juices** | 0 | 0 | 0 | 0 | 0 | 0 |
| **Alcoholic beverages** | 0 | 0 | 0 | 0 | 0 | 0 |
| **Sauces and salad dressings** | 0 | 0 | 0 | 0 | 0 | 0 |
| **Butter** | 0 | 0 | 0 | 0 | 0 | 0 |
| **Other fats** | 0 | 0 | 0 | 0 | 0 | 0 |
| **Chocolate and sweets** | 0 | 0 | -0.03677 | -0.006 | -0.02274 | 0 |
| **Nuts** | -0.01115 | -0.04636 | 0 | 0 | 0 | 0 |
| **Fast food and snacks** | 0 | 0 | 0 | -0.02831 | -0.06203 | 0 |
| **Appetizer products** | 0 | 0 | 0 | 0 | -0.02531 | 0 |
| **Cakes** | 0 | 0 | 0 | -0.01686 | 0 | 0 |
| **Legumes** | -0.01666 | -0.00603 | 0 | 0 | 0 | 0 |

| **Supplementary Table 3 (cont).** Precision matrix presenting the partial pairwise correlations between food groups adjusted for the effects of all other food groups in the sample. | | | | | | |
| --- | --- | --- | --- | --- | --- | --- |
|  | **Fruit juices 100% pure** | **Vegetable juices** | **Alcoholic beverages** | **Sauces and salad dressings** | **Butter** | **Other fats** |
| **Fruits** | 0 | 0 | 0 | 0 | 0 | 0 |
| **Charcuteries** | 0 | 0 | -0.09016 | -0.00906 | -0.0055 | 0 |
| **Vegetables** | 0 | 0 | 0 | 0 | 0 | -0.0309 |
| **Fish and seafood** | 0 | 0 | -0.02579 | 0 | 0 | 0 |
| **Fish charcuteries** | 0 | 0 | 0 | 0 | 0 | 0 |
| **Plant-based charcuteries** | 0 | 0 | 0 | 0 | 0 | 0 |
| **Pork and poultry hams** | 0 | 0 | 0 | 0 | 0 | 0 |
| **Meats** | 0 | 0 | -0.01442 | -0.02383 | 0 | 0 |
| **Poultry** | 0 | 0 | 0 | 0 | 0 | 0 |
| **Offals** | 0 | 0 | 0 | 0 | 0 | 0 |
| **Protein substitutes** | 0 | 0 | 0 | 0 | 0 | 0 |
| **Eggs** | 0 | 0 | 0 | 0 | 0 | -0.0277 |
| **Milk** | 0 | 0 | 0 | 0 | 0 | 0 |
| **Dairy substitutes** | 0 | 0 | 0 | 0 | 0.012138 | 0 |
| **Cheese** | 0 | 0 | -0.05664 | -0.00628 | -0.05687 | 0 |
| **Yoghurts** | 0 | 0 | 0 | 0 | 0 | 0 |
| **Dairy-based desserts** | 0 | 0 | 0 | 0 | 0 | 0 |
| **Plant-based dairy desserts** | 0 | 0 | 0 | 0 | 0 | 0 |
| **Starchy foods and tubers** | 0 | 0 | 0 | 0 | -0.01944 | -0.09166 |
| **Bread and crackers** | 0 | 0 | -7.4E-05 | -0.01263 | -0.13888 | 0 |
| **Whole-grain breads and crackers** | 0 | 0 | 0 | 0 | 0 | 0 |

| **Supplementary Table 3 (cont).** Precision matrix presenting the partial pairwise correlations between food groups adjusted for the effects of all other food groups in the sample. | | | | | | |
| --- | --- | --- | --- | --- | --- | --- |
|  | **Fruit juices 100% pure** | **Vegetable juices** | **Alcoholic beverages** | **Sauces and salad dressings** | **Butter** | **Other fats** |
| **Breakfast cereals and soft bars** | 0 | 0 | 0 | 0 | 0.036301 | 0 |
| **Pasta** | 0 | 0 | 0 | 0 | 0 | 0 |
| **Rice** | 0 | 0 | 0 | 0 | 0 | 0 |
| **Other cereal products** | 0 | 0 | 0 | 0 | 0 | 0 |
| **Whole-grain cereal products** | 0 | 0 | 0 | 0 | 0 | 0 |
| **Cookies** | 0 | 0 | 0 | 0 | 0 | 0 |
| **Pastries** | -0.00327 | 0 | 0 | 0 | 0 | 0 |
| **Non-alcoholic sugary beverages** | -0.01046 | 0 | 0 | 0 | 0 | 0 |
| **Non-alcoholic and non-sugary beverages** | 0 | 0 | 0 | 0 | 0 | 0 |
| **Fruit juices 100% pure** | 1.000124 | 0 | 0 | 0 | 0 | 0 |
| **Vegetable juices** | 0 | 1 | 0 | 0 | 0 | 0 |
| **Alcoholic beverages** | 0 | 0 | 1.037122 | -0.02515 | 0 | 0 |
| **Sauces and salad dressings** | 0 | 0 | -0.02515 | 1.001732 | 0 | 0 |
| **Butter** | 0 | 0 | 0 | 0 | 1.027495 | 0 |
| **Other fats** | 0 | 0 | 0 | 0 | 0 | 1.010147 |
| **Chocolate and sweets** | 0 | 0 | 0 | 0 | -0.02585 | 0 |
| **Nuts** | 0 | 0 | -0.01905 | 0 | 0 | -0.00783 |
| **Fast food and snacks** | 0 | 0 | 0 | 0 | 0 | 0 |
| **Appetizer products** | 0 | 0 | -0.15212 | 0 | 0 | 0 |
| **Cakes** | 0 | 0 | 0 | -0.00639 | 0 | 0 |
| **Legumes** | 0 | 0 | 0 | 0 | 0 | 0 |

| **Supplementary Table 3 (cont).** Precision matrix presenting the partial pairwise correlations between food groups adjusted for the effects of all other food groups in the sample. | | | | | | |
| --- | --- | --- | --- | --- | --- | --- |
|  | **Chocolate and sweets** | **Nuts** | **Fast food and snacks** | **Appetizer products** | **Cakes** | **Legumes** |
| **Fruits** | 0 | -0.05473 | 0 | 0 | 0 | 0 |
| **Charcuteries** | 0 | 0 | 0 | -0.02263 | -0.00864 | 0 |
| **Vegetables** | 0 | -0.05438 | 0.092614 | 0 | 0 | -0.00812 |
| **Fish and seafood** | 0 | 0 | 0 | 0 | 0 | 0 |
| **Fish charcuteries** | 0 | 0 | 0 | 0 | 0 | 0 |
| **Plant-based charcuteries** | 0 | 0 | 0 | 0 | 0 | 0 |
| **Pork and poultry hams** | 0 | 0 | 0 | 0 | 0 | 0 |
| **Meats** | 0 | 0 | 0 | 0 | 0 | 0 |
| **Poultry** | 0 | 0 | 0 | 0 | 0 | 0 |
| **Offals** | 0 | 0 | 0 | 0 | 0 | 0 |
| **Protein substitutes** | 0 | -0.0452 | 0 | 0 | 0 | -0.0209 |
| **Eggs** | 0 | 0 | 0 | 0 | 0 | 0 |
| **Milk** | 0 | 0.00328 | 0 | 0 | 0 | 0 |
| **Dairy substitutes** | 0 | -0.10735 | 0 | 0 | 0 | -0.02281 |
| **Cheese** | -0.00224 | 0 | 0 | 0 | 0 | 0 |
| **Yoghurts** | 0 | 0 | 0 | 0 | 0 | 0 |
| **Dairy-based desserts** | -0.01263 | 0 | 0 | 0 | 0 | 0 |
| **Plant-based dairy desserts** | 0 | 0 | 0 | 0 | 0 | 0 |
| **Starchy foods and tubers** | 0 | 0 | 0 | 0 | 0 | 0 |
| **Bread and crackers** | -0.05047 | 0 | 0 | 0 | 0 | 0 |
| **Whole-grain breads and crackers** | 0 | -0.03214 | 0 | 0 | 0 | 0 |

| **Supplementary Table 3 (cont).** Precision matrix presenting the partial pairwise correlations between food groups adjusted for the effects of all other food groups in the sample. | | | | | | |
| --- | --- | --- | --- | --- | --- | --- |
|  | **Chocolate and sweets** | **Nuts** | **Fast food and snacks** | **Appetizer products** | **Cakes** | **Legumes** |
| **Breakfast cereals and soft bars** | 0 | 0 | 0 | 0 | 0 | 0 |
| **Pasta** | 0 | 0 | 0 | 0 | 0 | 0 |
| **Rice** | 0 | 0 | 0 | 0 | 0 | 0 |
| **Other cereal products** | 0 | -0.01115 | 0 | 0 | 0 | -0.01666 |
| **Whole-grain cereal products** | 0 | -0.04636 | 0 | 0 | 0 | -0.00603 |
| **Cookies** | -0.03677 | 0 | 0 | 0 | 0 | 0 |
| **Pastries** | -0.006 | 0 | -0.02831 | 0 | -0.01686 | 0 |
| **Non-alcoholic sugary beverages** | -0.02274 | 0 | -0.06203 | -0.02531 | 0 | 0 |
| **Non-alcoholic and non-sugary beverages** | 0 | 0 | 0 | 0 | 0 | 0 |
| **Fruit juices 100% pure** | 0 | 0 | 0 | 0 | 0 | 0 |
| **Vegetable juices** | 0 | 0 | 0 | 0 | 0 | 0 |
| **Alcoholic beverages** | 0 | -0.01905 | 0 | -0.15212 | 0 | 0 |
| **Sauces and salad dressings** | 0 | 0 | 0 | 0 | -0.00639 | 0 |
| **Butter** | -0.02585 | 0 | 0 | 0 | 0 | 0 |
| **Other fats** | 0 | -0.00783 | 0 | 0 | 0 | 0 |
| **Chocolate and sweets** | 1.006844 | 0 | 0 | 0 | -0.03325 | 0 |
| **Nuts** | 0 | 1.03105 | 0 | 0 | 0 | -0.02965 |
| **Fast food and snacks** | 0 | 0 | 1.015085 | -0.0036 | -0.01949 | 0 |
| **Appetizer products** | 0 | 0 | -0.0036 | 1.02541 | -0.03095 | 0 |
| **Cakes** | -0.03325 | 0 | -0.01949 | -0.03095 | 1.002914 | 0 |
| **Legumes** | 0 | -0.02965 | 0 | 0 | 0 | 1.002848 |

| **Supplementary Table 4**. Pearson correlations between dietary pattern network scores. | | | | | |
| --- | --- | --- | --- | --- | --- |
|  | **Community 1 “Appetizers”** | **Community 2 “Breakfast”** | **Community 3 “Sweets and snacks”** | **Community 4 “Plant-based”** | **Community 5 “Healthy”** |
|  | **Pearson correlations** | | | | |
| **Community 1 “Appetizers”** |  |  |  |  |  |
| **Community 2 “Breakfast”** | -0.04 |  |  |  |  |
| **Community 3 “Sweets and snacks”** | 0.03 | 0.06 |  |  |  |
| **Community 4 “Plant-based”** | -0.15 | -0.08 | -0.1 |  |  |
| **Community 5 “Healthy”** | -0.05 | -0.1 | -0.27 | 0.13 |  |
| All Pearson correlations were significant. | | | | | |
| *Community 1 “Appetizers”* score includes 11 food groups: Alcoholic beverages, appetizer products, breads and crackers, butter, charcuteries, cheese, eggs, meats, other fats, sauces and salad dressings, starchy foods and tubers. | | | | | |
| *Community 2 “Breakfast”* score includes two food groups: Breakfast cereals and soft bars, milk. | | | | | |
| *Community 3 “Sweets and snacks”* score includes eight food groups: Cakes, chocolate and sweets, cookies, dairy-based desserts, fast food and snacks, fruit juices 100% pure, non-alcoholic sugary beverages, pastries. | | | | | |
| *Community 4 “Plant-based”* score includes nine food groups: Dairy substitutes, legumes, nuts, other cereal products, pasta, plant-based charcuteries, plant-based dairy desserts, protein substitutes, whole-grain cereal products. | | | | | |
| *Community 5 “Healthy”* score includes nine food groups: Fish and seafood, fruits, non-alcoholic and non-sugary beverages, pork and poultry hams, poultry, rice, vegetables, whole-grain breads and crackers, yoghurts. | | | | | |

| **Supplementary Table 5**. Means of dietary pattern network scores in the study sample and within subgroups of the sample. | | | | | | | | | | | | | |
| --- | --- | --- | --- | --- | --- | --- | --- | --- | --- | --- | --- | --- | --- |
| **Characteristic** | | **Number of participants** | **Community 1 “Appetizers”** | **Community 2 “Breakfast”** | | | **Community 3 “Sweets and snacks”** | **Community 4 “Plant-based”** | | | **Community 5 “Healthy”** | |  |
|  |  |  | Mean (Q1-Q3) | | | | | | | | | |  |
| **Total sample** | | 99 362 | 12.2 (7.3-15.4) | | 1.6 (0-2.4) | 5.8 (2.8-7.2) | | | 2.2 (0.4-2.1) | 41.9 (30.4-51.4) | | |  |
| **Sex** |  |  |  | |  |  | | |  |  | | |  |
|  | Men | 20 932 | 18.0 (11.1-23.0) | | 1.8 (0-2.8) | 6.6 (3.1-8.3) | | | 2.5 (0.5-2.4) | 41.8 (30.4-51.1) | | |  |
|  | Women | 78 430 | 10.7 (6.7-13.6) | | 2.1 (0-2.3) | 5.5 (2.7-7.0) | | | 2.2 (0.4-2.1) | 41.9 (30.4-51.5) | | |  |
| **Age groups (years)** | |  |  | |  |  | | |  |  | | |  |
|  | ≤ 25 | 14 607 | 10.2 (6.0-12.7) | | 2.1 (0.1-3.5) | 8.4 (4.2-10.8) | | | 2.0 (0.4-1.8) | 33.2 (22.1-41.6) | | |  |
|  | 26-50 | 51 867 | 12.0 (7.3-15.2) | | 1.6 (0-2.4) | 6.0 (3.1-7.6) | | | 2.3 (0.4-2.2) | 41.7 (30.3-50.8) | | |  |
|  | 51-65 | 27 724 | 13.3 (7.9-16.8) | | 1.3 (0-1.7) | 4.2 (2.2-5.3) | | | 2.3 (0.4-2.3) | 46.3 (35.6-55.4) | | |  |
|  | 66-80 | 5079 | 14.1 (8.4-18.2) | | 1.4 (0-2.1) | 3.6 (1.8-4.7) | | | 2.1 (0.4-2.2) | 45.2 (35.3-53.8) | | |  |
|  | >80 | 85 | 14.4 (8.9-19.6) | | 2.0 (0.1-3.5) | 3.2 (1.9-4.2) | | | 1.8 (0.3-1.7) | 42.5 (33.7-47.4) | | |  |
| **Smoking status** | |  |  | |  |  | | |  |  | | |  |
|  | Smoker | 16 281 | 14.2 (8.2-17.9) | | 1.4 (0-2.0) | 6.4 (2.9-8.0) | | | 1.7 (0.3-1.7) | 37.8 (26.4-47.2) | | |  |
|  | Former smoker | 32 602 | 13.2 (7.8-16.7) | | 1.4 (0-2.0) | 5.0 (2.4-6.3) | | | 2.4 (0.4-2.3) | 44.4 (33.0-53.7) | | |  |
|  | Non-smoker | 50 479 | 11.0 (6.7-13.9) | | 1.8 (0-2.8) | 6.0 (3.0-7.6) | | | 2.3 (0.4-2.2) | 41.6 (30.1-51.1) | | |  |
| **Education level** | |  |  | |  |  | | |  |  | | |  |
|  | < high school degree | 17 773 | 13.2 (7.8-16.7) | | 1.5 (0-2.3) | 5.3 (2.4-6.4) | | | 2.0 (0.3-1.9) | 41.3 (30.0-50.9) | | |  |
|  | ≤ bachelor’s degree | 48 817 | 11.8 (7.0-14.8) | | 1.6 (0-2.5) | 6.0 (2.9-7.5) | | | 2.2 (0.4-2.1) | 40.9 (29.3-50.6) | | |  |
|  | ≥ bachelor’s degree | 32 772 | 12.4 (7.3-15.7) | | 1.6 (0-2.3) | 5.7 (2.9-7.2) | | | 2.5 (0.4-2.4) | 43.7 (32.4-52.8) | | |  |
| **Household monthly income (EUR)** | |  |  | |  |  | | |  |  | | |  |
|  | < 1100 | 6633 | 10.5 (5.9-13.3) | | 1.9 (0-3.1) | 7.2 (3.2-9.2) | | | 2.7 (0.4-2.5) | 36.4 (24.3-45.7) | | |  |
|  | 1100 - < 2300 | 23 169 | 11.6 (6.7-14.6) | | 1.6 (0-2.5) | 6.1 (2.8-7.7) | | | 2.3 (0.4-2.2) | 40.5 (28.6-50.2) | | |  |
|  | 2300 - < 4800 | 53 274 | 12.4 (7.5-15.6) | | 1.6 (0-2.3) | 5.7 (2.8-7.2) | | | 2.1 (0.4-2.1) | 42.1 (30.9-51.5) | | |  |
|  | ≥ 4800 | 16 286 | 13.1 (8.1-16.5)) | | 1.4 (0-2.1) | 5.1 (2.7-6.4) | | | 2.2 (0.4-2.2) | 45.2 (34.2-54.3) | | |  |
| **Physical activity level** | |  |  | |  |  | | |  |  | | |  |
|  | Low | 22 464 | 12.3 (7.5-15.4) | | 1.6 (0-2.6) | 6.1 (3.0-7.8) | | | 1.9 (0.4-1.9) | 38.4 (27.6-47.3) | | |  |
|  | Moderate | 45 679 | 11.9 (7.1-15.0) | | 1.6 (0-2.3) | 5.8 (2.8-7.3) | | | 2.2 (0.4-2.1) | 41.5 (30.3-51.0) | | |  |
|  | High | 31 219 | 12.6 (7.2-16.0) | | 1.6 (0-2.3) | 5.4 (2.5-6.8) | | | 2.5 (0.4-2.4) | 44.9 (33.0-54.9) | | |  |
| **Cardiovascular disease** | |  |  | |  |  | | |  |  | | |  |
|  | Incidence of CVD | 1878 | 15.1 (8.9-19.4) | | 1.5 (0-2.1) | 4.9 (2.5-6.1) | | | 2.0 (0.4-2.0) | 44.6 (33.8-54.0) | | |  |
|  | No incidence of CVD | 97 484 | 12.2 (7.2-15.3) | | 1.6 (0-2.4) | 5.8 (2.8-7.3) | | | 2.2 (0.4-2.1) | 41.8 (30.3-51.4) | | |  |
| Mean dietary pattern network scores were all significantly different within subgroups of the population, except for mean *Community 5 “Healthy”* score between men and women. CVD, cardiovascular disease. | | | | | | | | | | | |  |  |
| *Community 1 “Appetizers”* score includes 11 food groups: Alcoholic beverages, appetizer products, breads and crackers, butter, charcuteries, cheese, eggs, meats, other fats, sauces and salad dressings, starchy foods and tubers. | | | | | | | | | | | |  |  |
| *Community 2 “Breakfast”* score includes two food groups: Breakfast cereals and soft bars, milk. | | | | | | | | | | | |  |  |
| *Community 3 “Sweets and snacks”* score includes eight food groups: Cakes, chocolate and sweets, cookies, dairy-based desserts, fast food and snacks, fruit juices 100% pure, non-alcoholic sugary beverages, pastries. | | | | | | | | | | | |  |  |
| *Community 4 “Plant-based”* score includes nine food groups: Dairy substitutes, legumes, nuts, other cereal products, pasta, plant-based charcuteries, plant-based dairy desserts, protein substitutes, whole-grain cereal products. | | | | | | | | | | | |  |  |
| *Community 5 “Healthy”* score includes nine food groups: Fish and seafood, fruits, non-alcoholic and non-sugary beverages, pork and poultry hams, poultry, rice, vegetables, whole-grain breads and crackers, yoghurts. | | | | | | | | | | | |  |  |

| **Supplementary Table 6**. Multivariable associations between adherence to the dietary pattern (DP) network scores and cardiovascular disease incidence in a sample of the NutriNet-Santé cohort (n=99 362), models including all DP networks concurrently. | | | | | | | |
| --- | --- | --- | --- | --- | --- | --- | --- |
| **Exposure** | | **Hazard ratio (95%CI)** | | | | | **p trend** |
| **Quintiles** | | **1** | **2** | **3** | **4** | **5** |  |
| **Community 1 “Appetizers”** | |  |  |  |  |  |  |
|  | Number of participants | 19872 | 19873 | 19872 | 19873 | 19872 |  |
|  | Number of cases | 234 | 284 | 336 | 394 | 630 |  |
|  | Model 1 | 1.00 | 1.03 (0.86-1.23) | 1.05 (0.88-1.25) | 1.05 (0.88-1.25) | 1.25 (1.04-1.51) | 0.02 |
|  | Model 2 | 1.00 | 1.02 (0.96-1.22) | 1.02 (0.86-1.22) | 0.96 (0.80-1.14) | 0.96 (0.79-1.17) | 0.55 |
| **Community 2 “Breakfast”** | |  |  |  |  |  |  |
|  | Number of participants | 27430 | 12262 | 19925 | 19837 | 19908 |  |
|  | Number of cases | 494 | 271 | 406 | 364 | 343 |  |
|  | Model 1 | 1.00 | 1.03 (0.89-1.20) | 1.11 (0.97-1.27) | 1.10 (0.96-1.26) | 1.02 (0.88-1.17) | 0.48 |
|  | Model 2 | 1.00 | 1.01 (0.87-1.18) | 1.10 (0.96-1.26) | 1.08 (0.94-1.24) | 1.00 (0.86-1.15) | 0.60 |
| **Community 3 “Sweets and snacks”** | |  |  |  |  |  |  |
|  | Number of participants | 19872 | 19873 | 19872 | 19873 | 19872 |  |
|  | Number of cases | 439 | 436 | 409 | 349 | 245 |  |
|  | Model 1 | 1.00 | 1.02 (0.89-1.17) | 1.10 (0.96-1.27) | 1.18 (1.01-1.27) | 1.27 (1.06-1.53) | 0.005 |
|  | Model 2 | 1.00 | 1.05 (0.91-1.20) | 1.15 (1.00-1.33) | 1.23 (1.05-1.44) | 1.31 (1.09-1.57) | 0.0004 |
| **Community 4 “Plant-based”** | |  |  |  |  |  |  |
|  | Number of participants | 19872 | 19874 | 19871 | 19873 | 19872 |  |
|  | Number of cases | 347 | 370 | 378 | 433 | 350 |  |
|  | Model 1 | 1.00 | 1.07 (0.93-1.24) | 0.99 (0.85-1.14) | 1.10 (0.96-1.27) | 0.99 (0.84-1.15) | 0.87 |
|  | Model 2 | 1.00 | 1.05 (0.90-1.21) | 0.96 (0.83-1.12) | 1.09 (0.94-1.26) | 0.98 (0.84-1.15) | 0.87 |
| **Community 5 “Healthy”** | |  |  |  |  |  |  |
|  | Number of participants | 19872 | 19873 | 19872 | 19873 | 19872 |  |
|  | Number of cases | 225 | 382 | 392 | 429 | 450 |  |
|  | Model 1 | 1.00 | 1.08 (0.91-1.27) | 0.90 (0.76-1.06) | 0.89 (0.75-1.05) | 0.91 (0.77-1.08) | 0.02 |
|  | Model 2 | 1.00 | 1.13 (0.95-1.33) | 0.97 (0.82-1.15) | 0.98 (0.82-1.16) | 1.03 (0.86-1.23) | 0.62 |
| Model 1: Adjusted for energy and DP network scores. | | | | | | | |
| Model 2: Adjusted for energy, sex, household income, education level, marital status, employment status, smoking status, number of cigarettes packs per year and number of dietary records and DP network scores.  DP: dietary patterns. | | | | | | | |

| **Supplementary Table 7**. Multivariable associations between adherence to dietary pattern (DP) network scores and cardiovascular disease incidence in a sample of the NutriNet-Santé cohort (n=99 362) when adjusting for energy, potential confounders and diet quality indices. | | | | | | | |
| --- | --- | --- | --- | --- | --- | --- | --- |
| **Exposure** | | **Hazard ratio (95%CI)** | | | | | **p trend** |
| **Quintiles** | | **1** | **2** | **3** | **4** | **5** |  |
| **Community 1 “Appetizers”** | |  |  |  |  |  |  |
|  | Number of participants | 19872 | 19873 | 19872 | 19873 | 19872 |  |
|  | Number of cases | 234 | 284 | 336 | 394 | 630 |  |
|  | Adjusted for PNNS 2 score | 1.00 | 1.00 (0.84-1.19) | 0.97 (0.82-1.16) | 0.88 (0.74-1.05) | 0.82 (0.67-0.99) | 0.02 |
|  | Adjusted for %UPF | 1.00 | 1.03 (0.87-1.23) | 1.04 (0.88-1.23) | 0.98 (0.83-1.16) | 0.99 (0.82-1.19) | 0.17 |
| **Community 2 “Breakfast”** | |  |  |  |  |  |  |
|  | Number of participants | 27430 | 12262 | 19925 | 19837 | 19908 |  |
|  | Number of cases | 494 | 271 | 406 | 364 | 343 |  |
|  | Adjusted for PNNS 2 score | 1.00 | 1.02 (0.88-1.19) | 1.12 (0.98-1.27) | 1.09 (0.95-1.25) | 1.01 (0.88-1.16) | 0.49 |
|  | Adjusted for %UPF | 1.00 | 1.00 (0.86-1.17) | 1.08 (0.95-1.24) | 1.07 (0.93-1.22) | 1.00 (0.87-1.15) | 0.16 |
| **Community 3 “Sweets and snacks”** | |  |  |  |  |  |  |
|  | Number of participants | 19872 | 19873 | 19872 | 19873 | 19872 |  |
|  | Number of cases | 439 | 436 | 409 | 349 | 245 |  |
|  | Adjusted for PNNS 2 score | 1.00 | 1.05 (0.92-1.20) | 1.16 (1.01-1.33) | 1.25 (1.07-1.45) | 1.32 (1.11-1.57) | 0.0002 |
|  | Adjusted for %UPF | 1.00 | 1.02 (0.89-1.16) | 1.10 (0.95-1.27) | 1.16 (0.99-1.35) | 1.19 (0.98-1.43) | 0.02 |
| **Community 4 “Plant-based”** | |  |  |  |  |  |  |
|  | Number of participants | 19872 | 19874 | 19871 | 19873 | 19872 |  |
|  | Number of cases | 347 | 370 | 378 | 433 | 350 |  |
|  | Adjusted for PNNS 2 score | 1.00 | 1.06 (0.91-1.22) | 0.97 (0.84-1.12) | 1.10 (0.95-1.27) | 1.01 (0.86-1.18) | 0.70 |
|  | Adjusted for %UPF | 1.00 | 1.05 (0.91-1.22) | 0.96 (0.83-1.11) | 1.09 (0.94-1.26) | 0.98 (0.84-1.14) | 0.99 |
| **Community 5 “Healthy”** | |  |  |  |  |  |  |
|  | Number of participants | 19872 | 19873 | 19872 | 19873 | 19872 |  |
|  | Number of cases | 225 | 382 | 392 | 429 | 450 |  |
|  | Adjusted for PNNS 2 score | 1.00 | 1.13 (0.96-1.34) | 0.98 (0.83-1.17) | 1.00 (0.94-1.18) | 1.05 (0.88-1.25) | 0.73 |
|  | Adjusted for %UPF | 1.00 | 1.14 (0.96-1.34) | 0.99 (0.84-1.17) | 1.00 (0.85-1.18) | 1.05 (0.89-1.25) | 0.76 |
| **With mutual adjustment for the DP network scores*** | | | | | | | |
| **Community 1 “Appetizers”** | |  |  |  |  |  |  |
|  | Number of participants | 19872 | 19873 | 19872 | 19873 | 19872 |  |
|  | Number of cases | 234 | 284 | 336 | 394 | 630 |  |
|  | Adjusted for PNNS 2 score | 1.00 | 1.00 (0.84-1.20) | 0.99 (0.83-1.18) | 0.91 (0.76-1.09) | 0.87 (0.71-1.08) | 0.14 |
|  | Adjusted for %UPF | 1.00 | 1.03 (0.86-1.23) | 1.04 (0.88-1.24) | 0.99 (0.83-1.18) | 1.00 (0.83-1.22) | 0.90 |
| **Community 2 “Breakfast”** | |  |  |  |  |  |  |
|  | Number of participants | 27430 | 12262 | 19925 | 19837 | 19908 |  |
|  | Number of cases | 494 | 271 | 406 | 364 | 343 |  |
|  | Adjusted for PNNS 2 score | 1.00 | 1.02 (0.87-1.18) | 1.11 (0.97-1.26) | 1.09 (0.95-1.25) | 1.00 (0.87-1.16) | 0.52 |
|  | Adjusted for %UPF | 1.00 | 1.00 (0.86-1.17) | 1.09 (0.95-1.24) | 1.00 (0.86-1.15) | 1.00 (0.86-1.15) | 0.64 |
| **Community 3 “Sweets and snacks”** | |  |  |  |  |  |  |
|  | Number of participants | 19872 | 19873 | 19872 | 19873 | 19872 |  |
|  | Number of cases | 439 | 436 | 409 | 349 | 245 |  |
|  | Adjusted for PNNS 2 score | 1.00 | 1.05 (0.92-1.20) | 1.15 (1.00-1.33) | 1.22 (1.05-1.43) | 1.28 (1.07-1.54) | 0.0008 |
|  | Adjusted for %UPF | 1.00 | 1.01 (0.88-1.16) | 1.09 (0.95-1.14) | 1.15 (0.98-1.35) | 1.18 (0.97-1.44) | 0.03 |
| **Community 4 “Plant-based”** | |  |  |  |  |  |  |
|  | Number of participants | 19872 | 19874 | 19871 | 19873 | 19872 |  |
|  | Number of cases | 347 | 370 | 378 | 433 | 350 |  |
|  | Adjusted for PNNS 2 score | 1.00 | 1.06 (0.91-1.22) | 0.98 (0.84-1.13) | 1.11 (0.96-1.29) | 1.02 (0.87-1.20) | 0.49 |
|  | Adjusted for %UPF | 1.00 | 1.05 (0.91-1.22) | 0.96 (0.83-1.12) | 1.09 (0.95-1.26) | 0.99 (0.84-1.16) | 0.80 |
| **Community 5 “Healthy”** | |  |  |  |  |  |  |
|  | Number of participants | 19872 | 19873 | 19872 | 19873 | 19872 |  |
|  | Number of cases | 225 | 382 | 392 | 429 | 450 |  |
|  | Adjusted for PNNS 2 score | 1.00 | 1.15 (0.97-1.36) | 1.00 (0.84-1.19) | 1.02 (0.86-1.21) | 1.09 (0.91-1.30) | 0.85 |
|  | Adjusted for %UPF | 1.00 | 1.14 (0.97-1.35) | 0.99 (0.84-1.17) | 1.01 (0.85-1.19) | 1.07 (0.90-1.28) | 0.97 |
| Adjusted for PNNS 2 score: Adjusted for model 2 in the main document (energy, sex, household income, education level, marital status, employment status, smoking status, number of cigarettes packs per year, number of dietary records) and the PNNS 2 score.  Adjusted for %UPF: Adjusted for model 2 in the main document (energy, sex, household income, education level, marital status, employment status, smoking status, number of cigarettes packs per year, number of dietary records) and the %UPF.  *Models are additionally adjusted for concurrent variations in DP network scores.  DP: dietary patterns.  PNNS 2 score: Programme National Nutrition Santé guidelines score 2.  %UPF: Represents the proportion of self-reported energy intake as ultra-processed foods (UPF) defined based on the NOVA food classification system. | | | | | | | |

| **Supplementary Table 8.** Multivariable associations between adherence to dietary pattern (DP) network scores and cardiovascular disease incidence when including only hard cardiovascular disease events (n = 98 061). | | | | | | | | |
| --- | --- | --- | --- | --- | --- | --- | --- | --- |
| **Exposure** | | **Hazard ratio (95%CI)** | | | | | **p trend** | |
| **Quintiles** | | **1** | **2** | **3** | **4** | **5** |  |  |
| **Community 1 “Appetizers”** | |  |  |  |  |  |  | |
|  | Number of participants | 19612 | 19612 | 19613 | 19612 | 19612 |  | |
|  | Number of cases | 55 | 78 | 109 | 130 | 205 |  | |
|  | Model 1 | 1.00 | 1.19 (0.84-1.68) | 1.40 (1.01-1.94) | 1.38 (1.00-1.90) | 1.47 (1.05-2.05) | 0.02 | |
|  | Model 2 | 1.00 | 1.16 (0.82-1.64) | 1.32 (0.95-1.83) | 1.19 (0.85-1.65) | 1.02 (0.72-1.44) | 0.78 | |
| **Community 2 “Breakfast”** | |  |  |  |  |  |  | |
|  | Number of participants | 27073 | 12076 | 19688 | 19552 | 19672 |  | |
|  | Number of cases | 137 | 85 | 125 | 123 | 107 |  | |
|  | Model 1 | 1.00 | 1.16 (0.89-1.52) | 1.20 (0.94-1.52) | 1.31 (1.03-1.67) | 1.10 (0.85-1.42) | 0.20 | |
|  | Model 2 | 1.00 | 1.16 (0.88-1.53) | 1.23 (0.96-1.57) | 1.31 (1.03-1.68) | 1.08 (0.84-1.40) | 0.25 | |
| **Community 3 “Sweets and snacks”** | |  |  |  |  |  |  | |
|  | Number of participants | 19612 | 19612 | 19613 | 19613 | 19611 |  | |
|  | Number of cases | 131 | 136 | 131 | 99 | 80 |  | |
|  | Model 1 | 1.00 | 1.08 (0.85-1.37) | 1.19 (0.93-1.53) | 1.13 (0.86-1.49) | 1.42 (1.05-1.94) | 0.04 | |
|  | Model 2 | 1.00 | 1.09 (0.86-1.40) | 1.26 (0.98-1.62) | 1.20 (0.91-1.59) | 1.50 (1.10-2.05) | 0.01 | |
| **Community 4 “Plant-based”** | |  |  |  |  |  |  | |
|  | Number of participants | 19612 | 19612 | 19613 | 19612 | 19612 |  | |
|  | Number of cases | 120 | 121 | 101 | 129 | 106 |  | |
|  | Model 1 | 1.00 | 1.01 (0.79-1.30) | 0.73 (0.56-0.96) | 0.89 (0.69-1.14) | 0.77 (0.59-1.00) | 0.03 | |
|  | Model 2 | 1.00 | 0.99 (0.77-1.28) | 0.72 (0.55-0.94) | 0.90 (0.70-1.16) | 0.83 (0.64-1.08) | 0.12 | |
| **Community 5 “Healthy”** | |  |  |  |  |  |  | |
|  | Number of participants | 19612 | 19612 | 19613 | 19612 | 19612 |  | |
|  | Number of cases | 69 | 121 | 109 | 136 | 142 |  | |
|  | Model 1 | 1.00 | 1.05 (0.78-1.42) | 0.74 (0.55-1.01) | 0.81 (0.60-1.08) | 0.79 (0.59-1.06) | 0.02 | |
|  | Model 2 | 1.00 | 1.13 (0.84-1.52) | 0.84 (0.62-1.14) | 0.96 (0.71-1.29) | 1.00 (0.74-1.35) | 0.63 | |
| **With mutual adjustment for the DP network scores*** | | | | | | | | |
| **Community 1 “Appetizers”** | |  |  |  |  |  |  | |
|  | Number of participants | 19612 | 19612 | 19613 | 19612 | 19612 |  | |
|  | Number of cases | 55 | 78 | 109 | 130 | 205 |  | |
|  | Model 1 | 1.00 | 1.17 (0.83-1.66) | 1.38 (0.99-1.93) | 1.35 (0.97-1.89) | 1.45 (1.01-2.08) | 0.05 | |
|  | Model 2 | 1.00 | 1.16 (0.82-1.64) | 1.32 (0.95-1.85) | 1.20 (0.85-1.68) | 1.05 (0.73-1.53) | 0.95 | |
| **Community 2 “Breakfast”** | |  |  |  |  |  |  | |
|  | Number of participants | 27073 | 12076 | 19688 | 19552 | 19672 |  | |
|  | Number of cases | 137 | 85 | 125 | 123 | 107 |  | |
|  | Model 1 | 1.00 | 1.16 (0.88-1.52) | 1.23 (0.96-1.57) | 1.33 (1.04-1.70) | 1.10 (0.85-1.42) | 0.19 | |
|  | Model 2 | 1.00 | 1.15 (0.87-1.51) | 1.22 (0.96-1.56) | 1.31 (1.03-1.68) | 1.06 (0.82-1.38) | 0.27 | |
| **Community 3 “Sweets and snacks”** | |  |  |  |  |  |  | |
|  | Number of participants | 19612 | 19612 | 19613 | 19613 | 19611 |  | |
|  | Number of cases | 131 | 136 | 131 | 99 | 80 |  | |
|  | Model 1 | 1.00 | 1.05 (0.83-1.34) | 1.17 (0.91-1.51) | 1.10 (0.83-1.47) | 1.40 (1.01-1.94) | 0.06 | |
|  | Model 2 | 1.00 | 1.07 (0.84-1.37) | 1.23 (0.95-1.59) | 1.15 (0.86-1.53) | 1.43 (1.03-1.98) | 0.02 | |
| **Community 4 “Plant-based”** | |  |  |  |  |  |  | |
|  | Number of participants | 19612 | 19612 | 19613 | 19612 | 19612 |  | |
|  | Number of cases | 120 | 121 | 101 | 129 | 106 |  | |
|  | Model 1 | 1.00 | 1.01 (0.78-1.30) | 0.75 (0.57-0.98) | 0.93 (0.72-1.20) | 0.86 (0.65-1.13) | 0.25 | |
|  | Model 2 | 1.00 | 0.99 (0.77-1.28) | 0.72 (0.55-0.95) | 0.91 (0.71-1.18) | 0.86 (0.65-1.13) | 0.26 | |
| **Community 5 “Healthy”** | |  |  |  |  |  |  | |
|  | Number of participants | 19612 | 19612 | 19613 | 19612 | 19612 |  | |
|  | Number of cases | 69 | 121 | 109 | 136 | 142 |  | |
|  | Model 1 | 1.00 | 1.09 (0.81-1.47) | 0.78 (0.57-1.06) | 0.87 (0.65-1.18) | 0.90 (0.66-1.22) | 0.29 | |
|  | Model 2 | 1.00 | 1.15 (0.85-1.55) | 0.85 (0.62-1.16) | 0.98 (0.72-1.33) | 1.05 (0.77-1.45) | 0.89 | |
| Model 1: Adjusted for energy. | | | | | | | |  |
| Model 2: Adjusted for energy, sex, household income, education level, marital status, employment status, smoking status, number of cigarettes packs per year and number of dietary records.  *Models are additionally mutually adjusted for the DP network scores.  DP: dietary patterns. | | | | | | | |  |

| **Supplementary Table 9**. Multivariable associations between adherence to dietary pattern (DP) network scores and cardiovascular disease incidence when excluding participants with prevalent type 2 diabetes, hypertension and dyslipidemia (n = 85 049). | | | | | | | | |
| --- | --- | --- | --- | --- | --- | --- | --- | --- |
| **Exposure** | | **Hazard ratio (95%CI)** | | | | | **p trend** | |
| **Quintiles** | | **1** | **2** | **3** | **4** | **5** |  |  |
| **Community 1 “Appetizers”** | |  |  |  |  |  |  | |
|  | Number of participants | 17009 | 17010 | 17010 | 17010 | 17010 |  | |
|  | Number of cases | 156 | 188 | 220 | 214 | 354 |  | |
|  | Model 1 | 1.00 | 1.06 (0.86-1.31) | 1.08 (0.88-1.33) | 0.90 (0.73-1.11) | 1.14 (0.92-1.42) | 0.63 | |
|  | Model 2 | 1.00 | 1.05 (0.85-1.30) | 1.06 (0.86-1.31) | 0.85 (0.68-1.05) | 0.92 (0.73-1.15) | 0.12 | |
| **Community 2 “Breakfast”** | |  |  |  |  |  |  | |
|  | Number of participants | 23286 | 10715 | 17035 | 17008 | 17005 |  | |
|  | Number of cases | 321 | 173 | 237 | 199 | 202 |  | |
|  | Model 1 | 1.00 | 0.99 (0.82-1.19) | 0.96 (0.81-1.13) | 0.93 (0.78-1.11) | 0.93 (0.78-1.11) | 0.33 | |
|  | Model 2 | 1.00 | 0.96 (0.80-1.16) | 0.96 (0.81-1.14) | 0.92 (0.77-1.09) | 0.91 (0.76-1.09) | 0.24 | |
| **Community 3 “Sweets and snacks”** | |  |  |  |  |  |  | |
|  | Number of participants | 17009 | 17010 | 17010 | 17010 | 17010 |  | |
|  | Number of cases | 254 | 251 | 261 | 207 | 159 |  | |
|  | Model 1 | 1.00 | 1.04 (0.87-1.24) | 1.23 (1.03-1.47) | 1.24 (1.02-1.51) | 1.45 (1.16-1.82) | 0.0004 | |
|  | Model 2 | 1.00 | 1.06 (0.89-1.26) | 1.28 (1.07-1.53) | 1.30 (1.06-1.58) | 1.51 (1.21-1.89) | <0.0001 | |
| **Community 4 “Plant-based”** | |  |  |  |  |  |  | |
|  | Number of participants | 17009 | 17010 | 17010 | 17010 | 17010 |  | |
|  | Number of cases | 207 | 218 | 232 | 250 | 225 |  | |
|  | Model 1 | 1.00 | 1.06 (0.88-1.29) | 1.01 (0.83-1.21) | 1.02 (0.85-1.23) | 0.94 (0.78-1.13) | 0.42 | |
|  | Model 2 | 1.00 | 1.05 (0.86-1.27) | 0.99 (0.82-1.19) | 1.03 (0.85-1.24) | 0.97 (0.80-1.17) | 0.69 | |
| **Community 5 “Healthy”** | |  |  |  |  |  |  | |
|  | Number of participants | 17009 | 17010 | 17010 | 17010 | 17010 |  | |
|  | Number of cases | 132 | 221 | 227 | 266 | 286 |  | |
|  | Model 1 | 1.00 | 1.05 (0.85-1.31) | 0.85 (0.69-1.06) | 0.87 (0.70-1.08) | 0.88 (0.71-1.09) | 0.06 | |
|  | Model 2 | 1.00 | 1.11 (0.89-1.38) | 0.93 (0.75-1.16) | 0.98 (0.79-1.22) | 1.03 (0.83-1.29) | 0.83 | |
| **With mutual adjustment for the DP network scores*** | | | | | | | | |
| **Community 1 “Appetizers”** | |  |  |  |  |  |  | |
|  | Number of participants | 17009 | 17010 | 17010 | 17010 | 17010 |  | |
|  | Number of cases | 156 | 188 | 220 | 214 | 354 |  | |
|  | Model 1 | 1.00 | 1.05 (0.84-1.30) | 1.07 (0.87-1.32) | 0.89 (0.72-1.11) | 1.16 (0.92-1.47) | 0.63 | |
|  | Model 2 | 1.00 | 1.05 (0.84-1.30) | 1.06 (0.85-1.31) | 0.85 (0.68-1.07) | 0.96 (0.75-1.23) | 0.27 | |
| **Community 2 “Breakfast”** | |  |  |  |  |  |  | |
|  | Number of participants | 23286 | 10715 | 17035 | 17008 | 17005 |  | |
|  | Number of cases | 321 | 173 | 237 | 199 | 202 |  | |
|  | Model 1 | 1.00 | 0.98 (0.82-1.18) | 0.97 (0.82-1.15) | 0.94 (0.78-1.12) | 0.93 (0.78-1.11) | 0.31 | |
|  | Model 2 | 1.00 | 0.96 (0.79-1.15) | 0.96 (0.81-1.14) | 0.91 (0.76-1.09) | 0.91 (0.76-1.09) | 0.22 | |
| **Community 3 “Sweets and snacks”** | |  |  |  |  |  |  | |
|  | Number of participants | 17009 | 17010 | 17010 | 17010 | 17010 |  | |
|  | Number of cases | 254 | 251 | 261 | 207 | 159 |  | |
|  | Model 1 | 1.00 | 1.04 (0.87-1.24) | 1.24 (1.03-1.48) | 1.24 (1.02-1.52) | 1.46 (1.15-1.85) | 0.001 | |
|  | Model 2 | 1.00 | 1.06 (0.89-1.27) | 1.28 (1.07-1.54) | 1.29 (1.05-1.58) | 1.50 (1.18-1.90) | 0.0002 | |
| **Community 4 “Plant-based”** | |  |  |  |  |  |  | |
|  | Number of participants | 17009 | 17010 | 17010 | 17010 | 17010 |  | |
|  | Number of cases | 207 | 218 | 232 | 250 | 225 |  | |
|  | Model 1 | 1.00 | 1.07 (0.88-1.29) | 1.02 (0.85-1.24) | 1.06 (0.88-1.27) | 1.01 (0.83-1.23) | 0.92 | |
|  | Model 2 | 1.00 | 1.04 (0.86-1.27) | 1.00 (0.82-1.20) | 1.04 (0.86-1.26) | 0.99 (0.81-1.21) | 0.87 | |
| **Community 5 “Healthy”** | |  |  |  |  |  |  | |
|  | Number of participants | 17009 | 17010 | 17010 | 17010 | 17010 |  | |
|  | Number of cases | 132 | 221 | 227 | 266 | 286 |  | |
|  | Model 1 | 1.00 | 1.09 (0.87-1.35) | 0.89 (0.72-1.11) | 0.93 (0.75-1.16) | 0.97 (0.78-1.21) | 0.28 | |
|  | Model 2 | 1.00 | 1.14 (0.91-1.41) | 0.96 (0.77-1.21) | 1.03 (0.82-1.28) | 1.10 (0.88-1.39) | 0.79 | |
| Model 1: Adjusted for energy | | | | | | | |  |
| Model 2: Adjusted for energy, sex, household income, education level, marital status, employment status, smoking status, number of cigarettes packs per year and number of dietary records.  *Models are additionally mutually adjusted for the DP network scores.  DP: dietary patterns. | | | | | | | |  |

**Supplementary Appendix 1.** Analytic code for the implementation of Gaussian Graphical Models and the Louvain algorithm.

**Introduction**

Description: The following code demonstrates the technical details of the Gaussian Graphical Model (GGM) and the Louvain algorithm employed in the analyses of this study. The objective was to identify dietary pattern networks using GGM and the Louvain algorithm. For complete objectives or more details, see paper.

Note: This code does not include data, codebook and complete analytic code for all objectives presented in the manuscript. Data described in the manuscript, codebook, and complete analytic code will be made available upon request pending application and approval.

Dataframes mentioned in the following code:

df_valid_temp: Dataframe of food group (n=42) consumption for all participants (n= 99 362) from the NutriNet-Santé study retained for the present analyses. Each of the 42 food groups is reported in grams/day.

df_valid_log_temp: Same dataframe as df_valid_temp, but with all food group variables transformed.


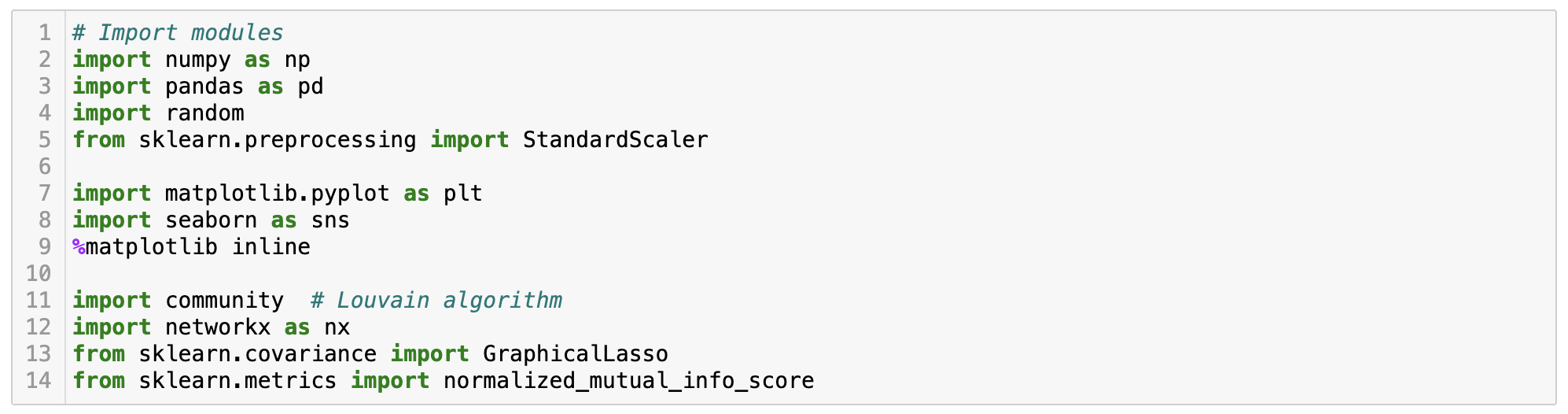


**Step 1: Gaussian Graphical Model (GGM)**

**1A. Generate sparse precision matrix:**

A sparse precision matrix outputs the pairwise partial correlations between all food groups (n=42), which are adjusted for the associations with and among all other variables. If the partial correlation between two variables is equal to zero, these variables are considered conditionally independent.


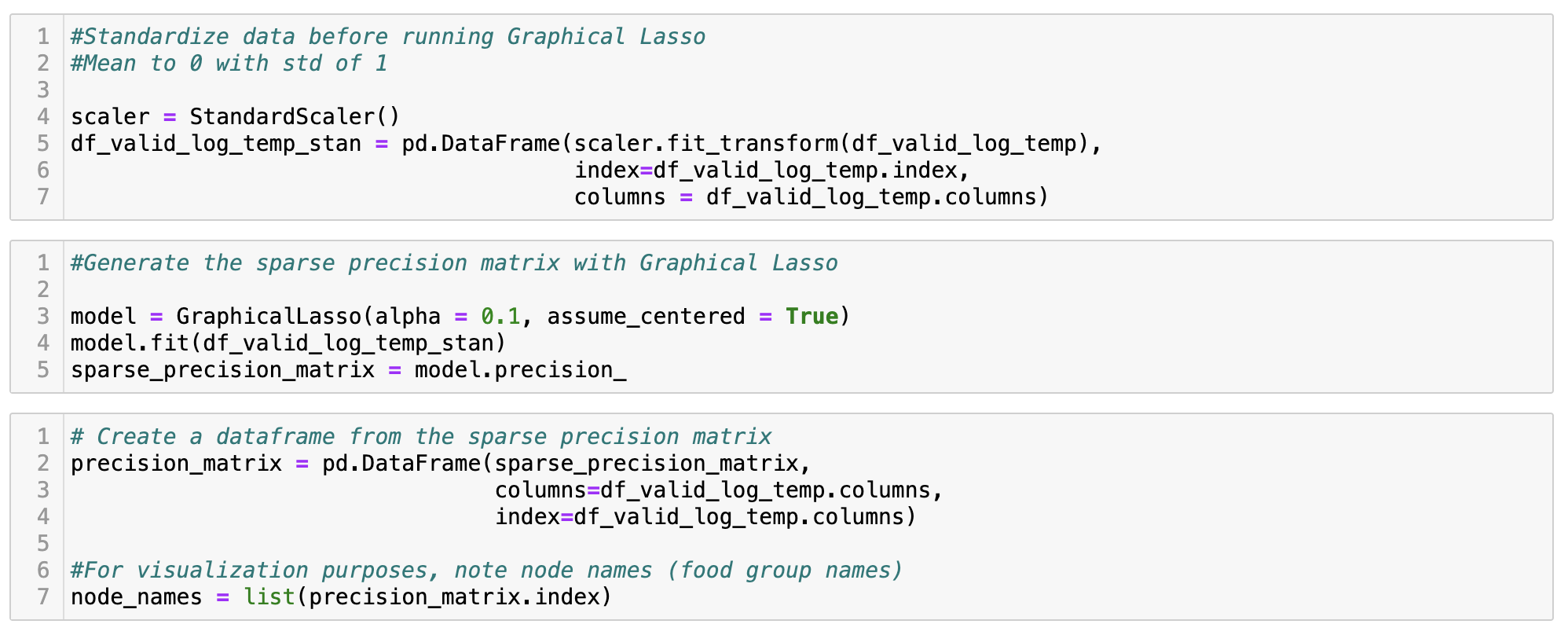


**1B. Initial GGM:**

GGMs are represented as undirected graphs, with each node corresponding to a variable and edges between nodes representing the partial correlation between two variables. In this GGM, all pairwise partial correlations are visualized.


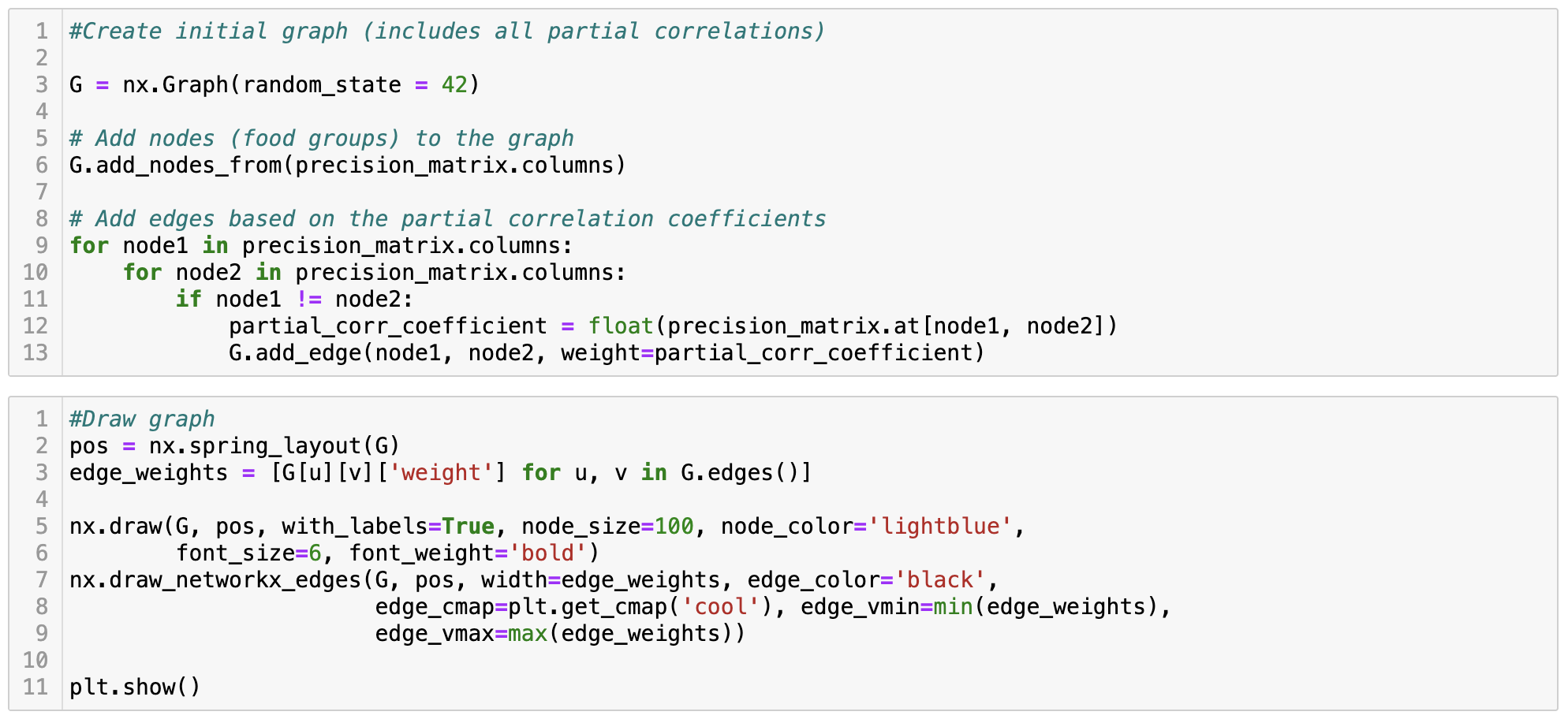


**Step 2: Louvain algorithm**

Once we have the GGM, we use the Louvain algorithm to identify communities in our GGM.

**2A. Run Louvain algorithm on the GGM:**

Run the Louvain algorithm (nx.algorithms.community.louvain_communities) on the GGM (G) to identify communities of food groups. Note that 5 communities are identified (with three food groups being excluded from the communities: Fish charcuteries, offals and vegetable juices)


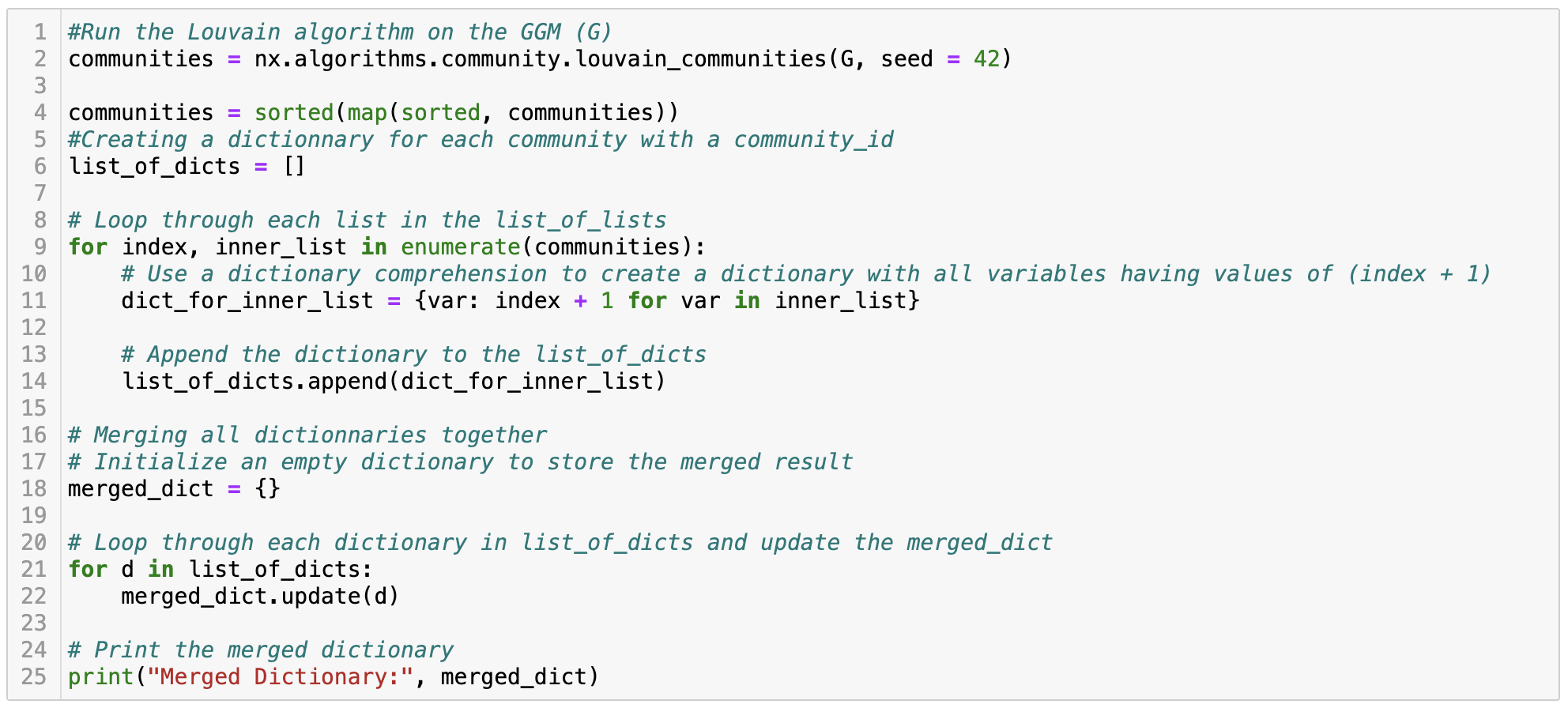


**2B. Run Louvain algorithm 99 separate times to evaluate stability of communities:**

Using the Normalized Mutual Information score (normalized_mutual_info_score), we verify the stability of the communities identified.


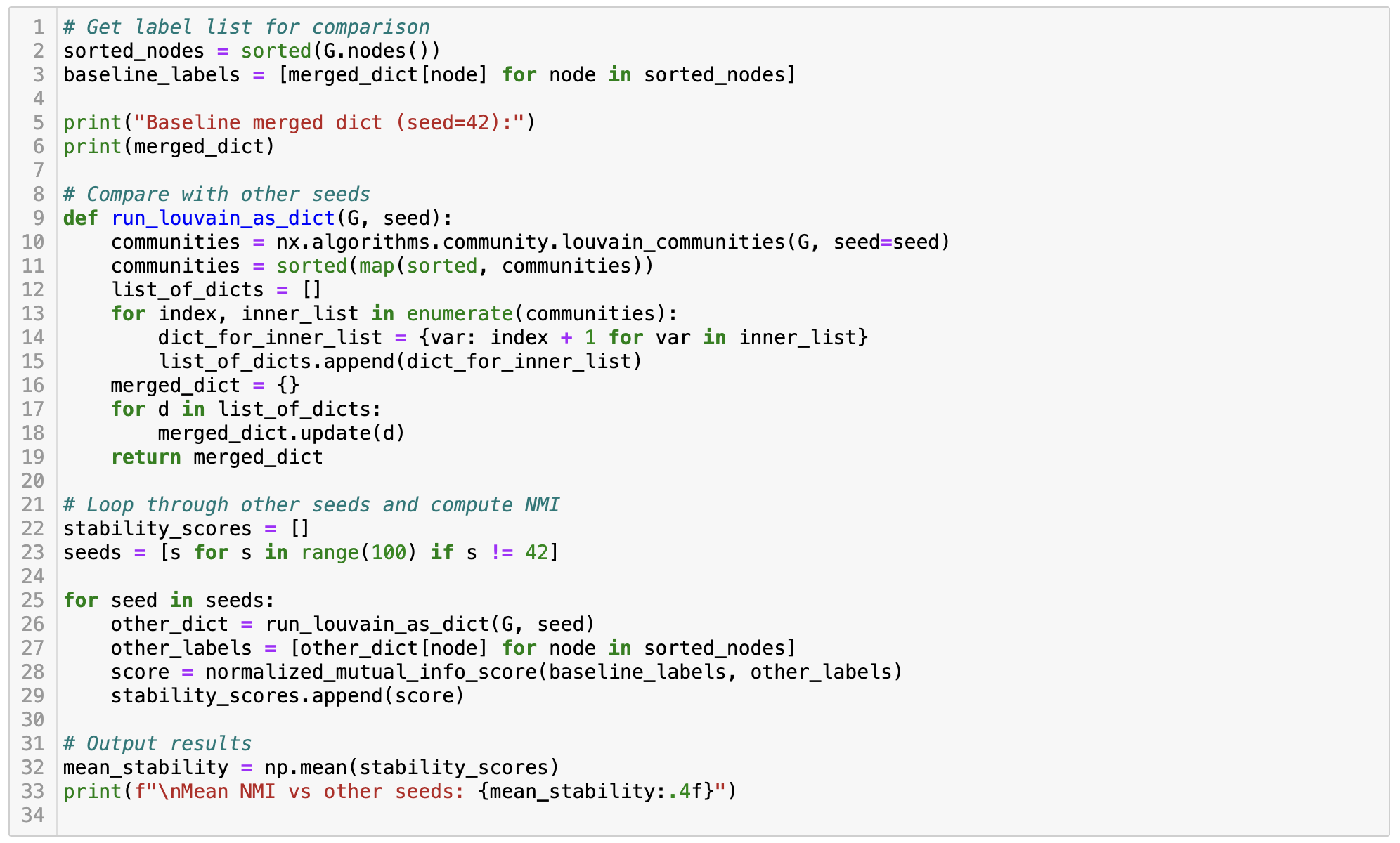


**2C. Graphical model with only non-zero edges:**

Meaning we exclude food groups with zero connections (Fish charcuteries, offals and vegetable juices) and visualize the GGM with the Louvain communities.


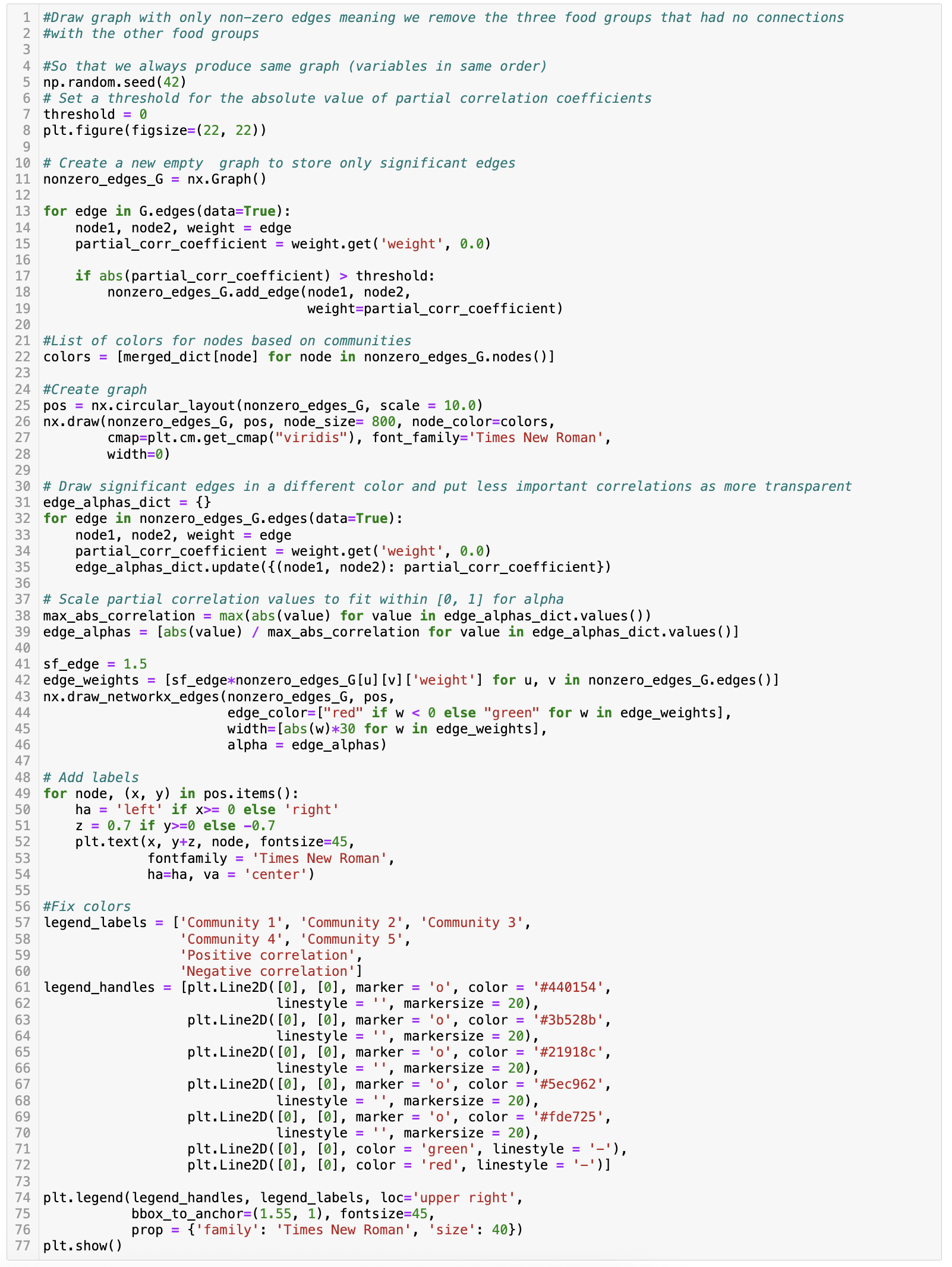


**Step 3: Generate dietary pattern scores**

**3A. Calculate eigenvector centrality:**

The eigenvector centrality is a measure of the relative importance, or “influence” of each food group within the network. A high eigenvector centrality score for a food group indicates that it has multiple and strong partial correlations with other food groups, thereby reflecting strong integration into the overall dietary pattern represented by the community.


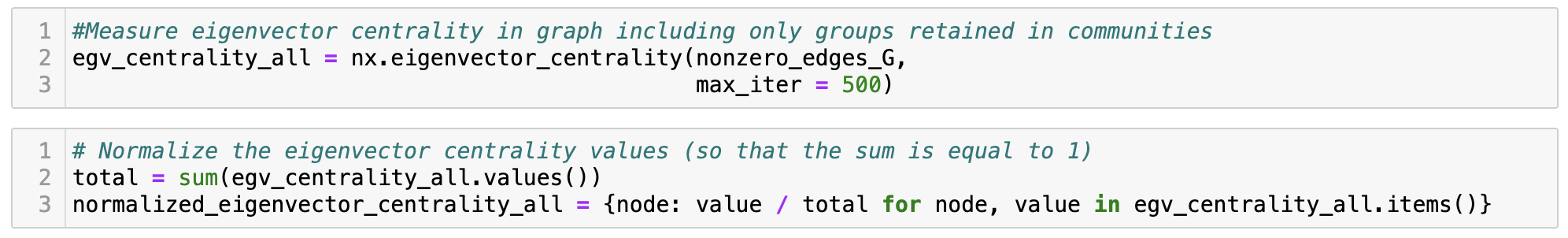


**3B. Calculate dietary pattern scores (one for each community) for each participant:**

Individual dietary pattern scores are calculated using the eigenvector centrality value assigned to each food group multiplied by the individual's daily intake of that food group (in grams). The dietary pattern network scores for each individual are calculated as the sum of each of these products within each community. The dietary pattern score is therefore influenced not only by eigenvector centrality of a given food group but also by the quantities of foods consumed by individuals. Higher dietary pattern network scores indicate a higher degree of adherence of a participant to that particular dietary pattern.


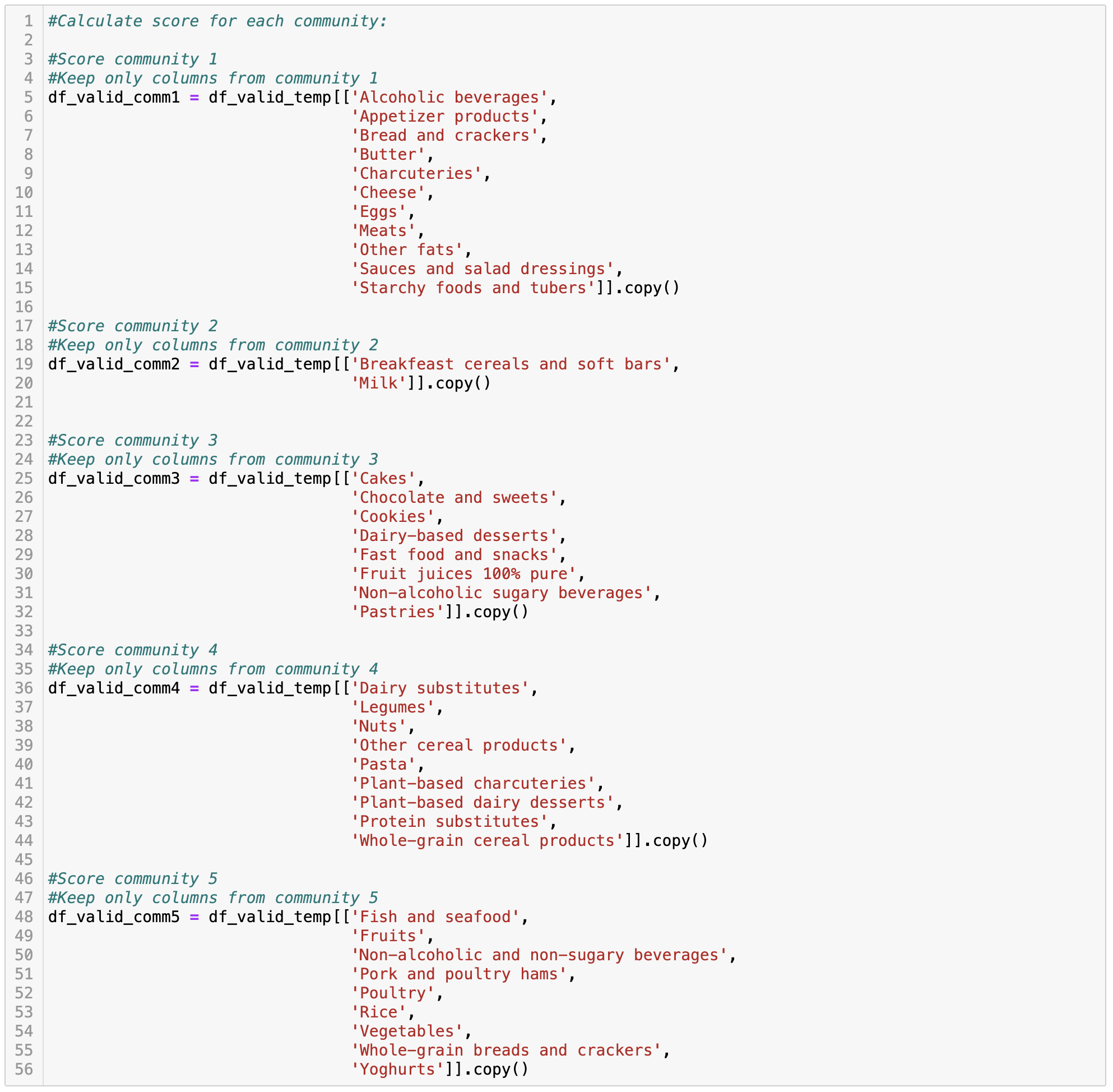


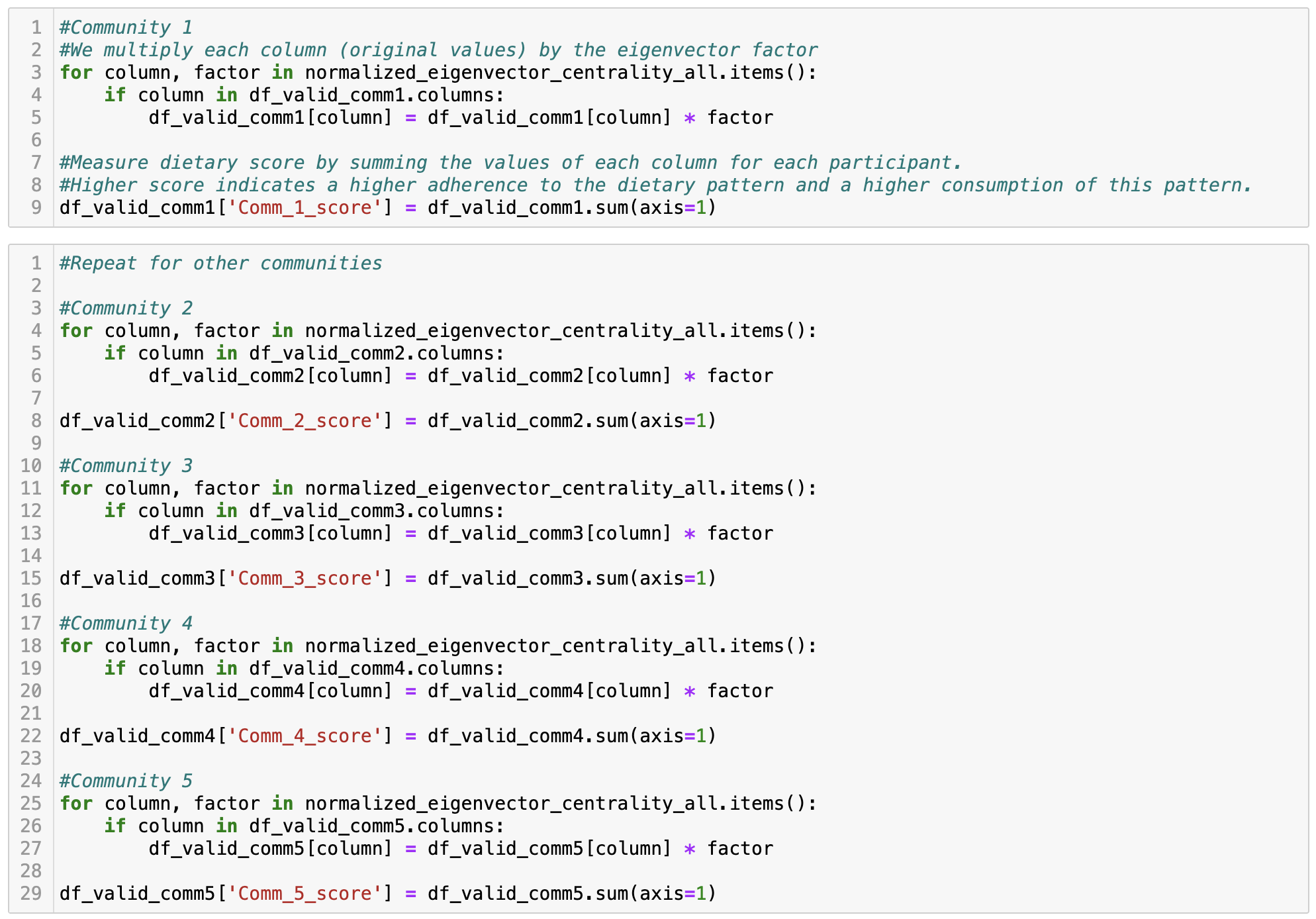

Supplement: multimedia component 1 [file mmc1.docx]
